# Effects of *N*-oxidation on the molecular and crystal structures and properties of isocinchomeronic acid, its metal complexes and their supramolecular architectures: experimental, CSD survey, solution and theoretical approaches†

**DOI:** 10.1039/c9ra05143k

**Published:** 2019-08-14

**Authors:** Zahra Hosseini-Hashemi, Masoud Mirzaei, Ameneh Jafari, Peyman Hosseinpour, Mohammad Yousefi, Antonio Frontera, Mahmoud Lari Dashtbayaz, Mojtaba Shamsipur, Mehdi Ardalani

**Affiliations:** Department of Chemistry, Faculty of Science, Ferdowsi University of Mashhad Mashhad Iran mirzaeesh@um.ac.ir; Department of Chemistry, Yadegar-e-Imam Khomeini (RAH) Shahr-e-Rey Branch, Islamic Azad University Tehran Iran myousefi50@hotmail.com; Department of Chemistry, Universitat de les Illes Balears Crta de Valldemossa km 7.5 07122 Palma de Mallorca (Baleares) Spain; Department of Economics and Administrative Sciences, Ferdowsi University of Mashhad Mashhad Iran; Department of Analytical Chemistry, Razi University Kermanshah Iran

## Abstract

Nine coordination complexes and polymer (M/L/X) based on Co, Ni, Zn, Cu (M), pyridine-*N*-oxide-2,5-dicarboxylic acid (H_2_pydco) (L) and either isonicotinamide (Ina), piperazine (pipz), 2,2′-bipyridine (bipy) and 1,10-phenanthroline (phen) (X) were synthesized and characterized by elemental analyses, infrared spectroscopy and single crystal X-ray diffraction. The resulting empirical formulae of the prepared complexes are [Co(H_2_O)_6_][Co(pydco)_2_(H_2_O)_2_]·2H_2_O (1), [M(pydco)(H_2_O)_4_]_2_ [M = Co (2), Ni (3), Zn (4)], [Co(pydco)(bipy)(H_2_O)_2_]·4H_2_O (5), [Co(pydco)(phen)(H_2_O)_2_]·5.135(H_2_O)·0.18(EtOH) (6), [Cu(Hpydco)(bipy)Cl]·2H_2_O (7), [Cu(Hpydco)(bipy)Cl]_2_·2H_2_O (8), and {[AgCu(H_2_O)_2_(phen)(pydco)]NO_3_}_*n*_ (9). With the exception of 9, which forms an extended structure *via* multiple coordination modes, all the complexes contain (H)pydco as a bidentate ligand coordinated to the metal ion *via* the *N*-oxide and the adjacent carboxylate group oxygen atom, creating a chelate ring. The metal centers exhibit either distorted octahedral (1–6) or square pyramidal (7–9) geometry. Our results demonstrate that, when acting cooperatively, non-covalent interactions such as X–H⋯O hydrogen bonds (X = O, N, C), C–O⋯π and π⋯π stacking represent driving forces for the selection of different three-dimensional structures. Moreover, in compounds 2–4, 1D supramolecular chains are formed where O⋯π–hole interactions are established, which unexpectedly involve the non-coordinated carboxylate group. The non-covalent interaction (NCI) plot index analysis reveals the existence of the O⋯π–hole interactions that have been evaluated using DFT calculations. The Cremer and Pople ring puckering parameters are also investigated. The complexation reactions of these molecules with M were investigated by solution studies. The stoichiometry of the most abundant species in the solution was very close to the corresponding crystals. Finally, the effect of *N*-oxidation on the geometry of complexes has been also studied using the Cambridge Structural Database. It shows that complexes containing *N*-oxidized H_2_pydc are very rare.

## Abstract 2

A series of new coordination complexes bearing H_2_pydco, an O-donor ligand, were synthesized and compared to corresponding complexes with H_2_pydc. The molecular and supramolecular structures were investigated using crystallography, solution phase and high level DFT studies.

## Graphical abstract

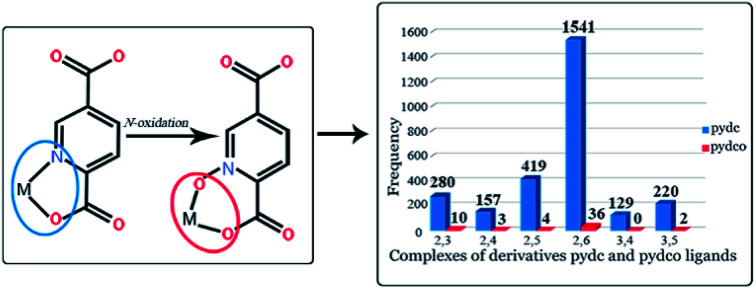

## Introduction

**Scheme 1:**
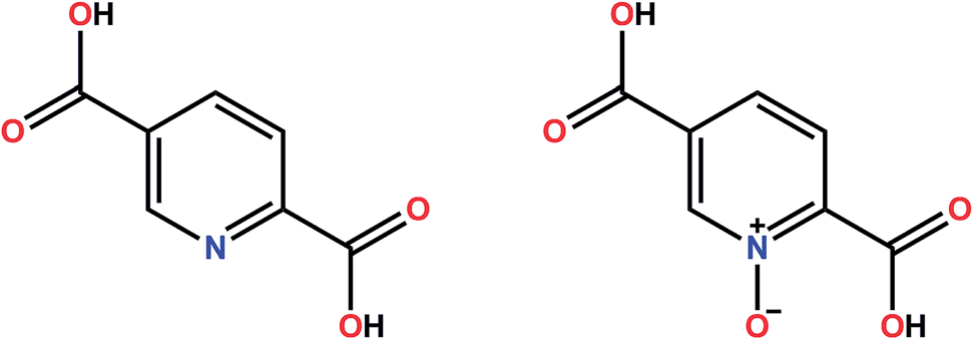
The structures of H_2_pydc and H_2_pydco.

Over the last few decades, considerable attention has been devoted to crystal engineering, which is the design and synthesis of solid-state molecular structures with desired properties, based on the understanding and exploitation of intermolecular interactions. A variety of non-covalent interactions including hydrogen bonding, π⋯π stacking, C–H⋯π and other interactions produce many interesting structures with 1D (pillar, chain or band), 2D (layer) and 3D (network) topologies.^1–3^ Intermolecular forces are considerably weaker than covalent bonds, so that supramolecular species are thermodynamically less stable, kinetically more labile and dynamically more flexible than molecules. Supramolecular chemistry is therefore concerned with weak bonds. In the production of supramolecular scaffolds several factors such as the type of metal, solvent, organic ligand and auxiliary ligand play crucial roles.^4^ Auxiliary ligands such as 2,2′-bipyridine and 1,10-phenanthroline are bidentate with two aromatic units each carrying an N-donor atom. The crystal structures formed by these ligands can display weak π⋯π interactions that are very important for the construction of multidimensional arrays.^5^ In recent years, researchers have been attracted to the many special properties and fascinating applications of complexes containing dicarboxylic acid and pyridine rings.^6^ These applications exist in many areas, such as catalysis,^7^ antibacterial activity,^8^ anticancer properties,^9^ aqueous solution chemistry,^10^ surface chemistry,^11^ magnetism^12,13^ and fluorescence.^14,15^ Our research group therefore set out to synthesize coordination complexes based on derivatives of pyridinedicarboxylic acids with a view to their significant applications.^16–20^ Among the structural isomers of 2,*n*-pyridinedicarboxylic acids (*n* = 3–6), H_2_pydc (isocinchomeronic acid or pyridine-2,5-dicarboxylic acid) is an appropriate candidate for constructing metallotectons due to its interesting structural features: it is a multidentate ligand containing N- and O-donor atoms; it has two carboxylates on opposite sides of the pyridine ring. These features confer the potential to construct higher-dimensional extended structures.^21,22^ The carboxylate groups display various coordination modes including monodentate (terminal and bridging) and bidentate (chelating and bridging).^23^ Meanwhile, 5-carboxylate has a stronger bridging capability than 2-carboxylate due to its strong electron-donating and electrostatic power.^24^*N*-oxidation of the pyridine ring adds a new coordination mode: whereas the nitrogen atom of the pyridine ring in H_2_pydc can donate one pair of electrons, the *N*-oxide group can donate two pairs and can therefore coordinate to a larger number of metal centers, resulting in a greater variety of bridging modes for H_2_pydco compared to H_2_pydc (see Scheme 1).^25–27^ We were inspired to synthesize the *N*-oxide form of the ligand by the work of Xiong *et al.* who demonstrated how *N*-oxide functionalization could greatly enhance the CO_2_ separation properties of isoreticular MOFs.^28^ Moreover, this family of pyridine *N*-oxides have been utilized as an anti-HIV agent, gas adsorbent, luminescent agent, *etc*.,^28–30^ which encouraged us to prepare new complexes with the pyridine-*N*-oxide-2,5-dicarboxylic acid. Our studies concur with previous investigations in showing that H_2_pydco tends to be an effective chelating and bridging ligand.^27^ Herein, we report the preparation of [Co(H_2_O)_6_][Co(pydco)_2_(H_2_O)_2_]·2H_2_O (1), [M(pydco)(H_2_O)_4_]_2_ [M = Co (2), Ni (3), and Zn (4)], [Co(pydco)(bipy)(H_2_O)_2_]·4H_2_O (5), [Co(pydco)(phen)(H_2_O)_2_]·5.135(H_2_O)·0.18(EtOH) (6), [Cu(Hpydco)(bipy)Cl]·2H_2_O (7), [Cu(Hpydco)(bipy)Cl]_2_·2H_2_O (8), and {[AgCu(H_2_O)_2_(phen)(pydco)]NO_3_}_*n*_ (9), where H_2_pydco = pyridine-*N*-oxide-2,5-dicarboxylic acid, Ina = isonicotinamide, pipz = piperazine, bipy = 2,2′-bipyridine and phen = 1,10-phenanthroline (see Scheme S1†). By means of DFT calculations (M06-2X-D/def2-TZVP), we have studied the importance of π–hole interactions involving the carboxylate group (as π–hole) donor in compounds 2–4 and the influence of the metal center (M = Co, Ni, Zn) on the interaction strength. Moreover, we have also performed the non-covalent interaction (NCI) index analysis in complexes 2–4 to confirm the existence of π–hole O⋯CO interactions and their interplay with H-bonding interactions. The complexation reactions of these supramolecular systems in aqueous solution were investigated by potentiometric pH titration method (solution studies) in order to characterize the stoichiometry of new species. The solution behavior of the investigated species, providing additional evidence of the interactions between adduct and metal ions, supporting the results obtained from the solid-state studies.

## Experimental

### General methods and materials

All chemicals and solvents were purchased from commercial sources and used without further purification. Melting points were determined using a Barnstead Electrothermal 9300 apparatus. IR spectra were recorded from KBr pellets in the 4000–600 cm^−1^ region using a Buck 500 IR spectrometer. Elemental analysis (CHN) was performed using a Thermo Finning Flash-1112 EA microanalyzer. The mass spectrum was scanned on a MS model CH7A Varian (EI, 70 eV). ^1^H NMR, and ^13^C{^1^H} NMR spectra were recorded in EtOD solution on a FT-NMR Bruker Avance III 300 MHz spectrometer, using TMS for ^1^H and ^13^C as the internal standard.

### X-ray structure determination and structure refinement

Single-crystal X-ray diffraction measurements were performed on a STOE IPDS-2T diffractometer with graphite monochromated Mo-Kα radiation. Single crystals were chosen using a polarizing microscope and were mounted on glass fibers for data collection. Cell constants and orientation matrices were obtained by least-squares refinement against setting angles for all reflections. Diffraction data were collected as a series of 1° *ω* scans and integrated using the Stoe X-AREA software package.^31^ Lorentz and polarization corrections were applied to the data and a numerical absorption correction was applied using X-RED^32^ and X-SHAPE.^33^ The structures were solved by direct methods^34^ and subsequent difference Fourier maps and then refined on *F*^2^ by full-matrix least-squares with anisotropic displacement parameters for all non-H atoms.^35^ The atomic scattering factors were taken from International Tables for X-ray Crystallography.^36^ All refinements were performed within the Stoe X-STEP32 structure evaluation package.^37^

### Synthesis of pyridine-*N*-oxide-2,5-dicarboxylic acid

H_2_pydc (1.003 g, 6 mmol) and a solution of Na_2_WO_4_·2H_2_O (0.065 g, 0.2 mmol) in hydrogen peroxide (30%, 9 mL) were heated at 90–100 °C with vigorous stirring for 50 min. Then an additional amount of hydrogen peroxide (30%, 22 mL) was added dropwise over a period of 2 h until all insoluble material had disappeared. After an additional 3 h of heating, the reaction mixture was allowed to stand for 24 h. The yellow crystalline solid was filtered off and dried in air. This product was obtained in 95% yield (based on H_2_pydc) (m.p. 198 °C). Anal. calcd for C_7_H_5_NO_5_: C 45.91; H 2.75; N 7.65%. Found: C 46.22; H 2.77; N 7.73%. IR bands (KBr pellet, cm^−1^): 3446–3081(br), 2634(m), 1725(s), 1643(m), 1612(m), 1513(m), 1402(s), 1231(s). MS (70 eV, EI): *m*/*z* (%) = 183 (45) [M]^+^, 182 (96) [M − 1]^+^, 138 (98) [M − COOH]^+^, 122 (100) [C_6_H_4_NO_2_]^+^, 94 (27) [C_5_H_4_NO]^+^, 78 (95) [C_4_H_5_N]^+^, 45 (85) [COOH]^+^. ^1^H NMR (300.81 MHz, EtOD, 296.6 K, TMS): *δ* 8.36 (dd, ^3^*J*_H–H_ = 8.4 Hz, ^4^*J*_H–H_ = 1.5 Hz, 1H, Ar-H_b_), 8.52 (d, ^3^*J*_H–H_ = 8.1 Hz, 1H, Ar-H_c_), 8.96 (d, ^4^*J*_H–H_ = 1.2 Hz, 1H, Ar-H_a_). ^13^C{^1^H} NMR (75.64 MHz, EtOD, 297.9 K, TMS): *δ* 129.08 (s), 131.55 (s), 133.81 (s), 138.60 (s), 139.88 (s), 160.95 (s), 162.70 (s). The MS, ^1^H NMR and ^13^C NMR spectra of H_2_pydco are given in Fig. S1–S3.†

### Syntheses of [Co(H_2_O)_6_][Co(pydco)_2_(H_2_O)_2_]·2H_2_O (1) and [Co(pydco)(H_2_O)_4_]_2_ (2)

A solution of H_2_pydco (0.183 g, 1 mmol) in ethanol–water (1 : 1; 20 mL) was added dropwise with stirring to a solution of Co(CH_3_COO)_2_·4H_2_O (0.249 g, 1 mmol) in water (30 mL) at 50 °C. A suspension formed immediately and the solution was stirred for 2 h at room temperature. Ina (0.122 g, 1 mmol) in ethanol (30 mL) was added dropwise to this suspension and was stirred for 1 h at 60 °C and then cooled to room temperature.^38^ Two types of crystals [orange (1) and pink (2)] were obtained by slow evaporation after one week. The final yields were 0.25 g for 1 (38.4%) and 0.08 g (26.8%) for 2 (based on Co). Data for 1: (m.p. 157 °C), Anal. calcd for C_14_H_26_Co_2_N_2_O_20_: C 25.47; H 3.97; N 4.24%. Found: C 25.58; H 3.89; N 4.31%. IR bands (KBr pellet, cm^−1^): 3372(br), 1662(m), 1611(s), 1390(s), 1352(m), 1217(m). Data for 2: (m.p. 177 °C), Anal. calcd for C_7_H_11_CoNO_9_: C 26.94; H 3.55; N 4.49%. Found: C 26.78; H 3.41; N 4.63%. IR bands (KBr pellet, cm^−1^): 3258(br), 1639(s), 1591(s), 1552(m), 1390(s), 1352(m), 1211(w).

### Synthesis of [Ni(pydco)(H_2_O)_4_]_2_ (3)

A solution of H_2_pydco (0.092 g, 0.5 mmol) in THF (10 mL) was added dropwise to a solution of pipz (0.043 g, 0.5 mmol) in THF (10 mL) and stirred at room temperature for 2 h. A solution of NiCl_2_·6H_2_O (0.119 g, 0.5 mmol) in water (15 mL) was added dropwise to the above solution and stirring was continued for 2 h at room temperature.^39,40^ After one-month, slow evaporation yielded green acicular crystals of 3 (m.p. > 300 °C) in *ca.* 43% yield based on Ni. Anal. calcd for C_7_H_11_NNiO_9_: C 26.96; H 3.56; N 4.49%. Found: C 26.64; H 3.63; N 4.39%. IR bands (KBr pellet, cm^−1^): 3362 (br), 1640(s), 1597(s), 1553(m), 1391(s), 1350(m), 1210(w).

### Synthesis of [Zn(pydco)(H_2_O)_4_]_2_ (4)

Preparation of 4 was similar to that of 3 except that Zn(NO_3_)_2_·6H_2_O (0.148 g, 0.5 mmol) was used instead of NiCl_2_·6H_2_O.^39,41^ Colourless platy crystals of compound 4 (m.p. > 300 °C) were obtained *in ca.* 54% yield based on Zn. Anal. calcd for C_7_H_11_NO_9_Zn: C 26.39; H 3.48; N 4.40%. Found: C 26.41; H 3.26; N 4.24%. IR bands (KBr pellet, cm^−1^): 3350(br), 1639(s), 1591(s), 1552(m), 1390(s), 1352(m), 1211(w).

### Synthesis of [Co(pydco)(bipy)(H_2_O)_2_]·4H_2_O (5)

A solution of bipy (0.031 g, 0.2 mmol) in ethanol–water (1 : 1; 3 mL) was added dropwise to a solution of H_2_pydco (0.037 g, 0.2 mmol) in ethanol–water (1 : 1; 10 mL) heated under reflux at 80–90 °C. After 1 h, a solution of Co(CH_3_COO)_2_·4H_2_O (0.049 g, 0.2 mmol) in ethanol–water (1 : 1; 3 mL) was added and refluxed for 6 h at 80–90 °C. After 10 days, orange blocky crystals of 5 (m.p. 177 °C) were obtained by slow evaporation *in ca.* 40% yield based on Co. Anal. calcd for C_17_H_23_CoN_3_O_11_: C, 40.49; H, 4.60; N, 8.33%. Found: C, 40.70; H, 4.35; N, 8.26%. IR (KBr pellet, cm^−1^): 3245(br), 1628(s), 1553(m), 1387(s), 1350(m), 1211(m).

### Synthesis of [Co(pydco)(phen)(H_2_O)_2_]·5.135(H_2_O)·0.18(EtOH) (6)

Complex 6 was synthesized following a procedure similar to that employed for the synthesis of complex 5 but replacing bipy with phen (0.039 g, 0.2 mmol). Orange blocky crystals of compound 6 (m.p. 212 °C) were obtained *in ca.* 48% yield based on Co. Anal. calcd for C_19.36_H_26.35_CoN_3_O_12.32_: C, 41.73; H, 4.76; N, 7.54%. Found: C, 41.43; H, 4.99; N, 7.32%. IR (KBr pellet, cm^−1^): 3316(br), 1632(s), 1519(m), 1394(s), 1352(s), 1214(m).

### Syntheses of [Cu(Hpydco)(bipy)Cl]·2H_2_O (7) and [Cu(Hpydco)(bipy)Cl]_2_·2H_2_O (8)

A solution of H_2_pydco (0.092 g, 0.5 mmol), bipy (0.078 g, 0.5 mmol) and CuCl_2_·2H_2_O (0.085 g, 0.5 mmol) in ethanol–water (1 : 1; 25 mL) was prepared and stirred at room temperature. After 15 min a suspension formed and the mixture was stirred for 4 h at room temperature. Two differently-colored acicular crystals [green (7) and blue (8)] were obtained by slow evaporation *in ca*. 52% yield (based on Cu) after 2 weeks. Data for 7 (m.p. 230 °C). Anal. calcd for C_17_H_16_ClCuN_3_O_7_: C 43.14; H 3.41; N 8.88%. Found: C 43.93; H 3.30; N 9.05%. IR bands (KBr pellet, cm^−1^): 3379(br), 3066(m), 1695(m), 1643(s), 1603(s), 1565(m), 1398(s), 1345(m), 1206(m).

Data for 8 (m.p. 244 °C). Anal. calcd for C_34_H_27_ClCu_2_N_6_O_12_: C 46.72; H 3.11; N 9.61%. Found: C 47.27; H 3.00; N 9.89%. IR bands (KBr pellet, cm^−1^): 3505(br), 3071(m), 1741(m), 1652(s), 1567(w), 1392(m), 1341(m), 1205(m).

### Synthesis of {[AgCu(H_2_O)_2_(phen)(pydco)]NO_3_}_*n*_ (9)

H_2_pydco (0.092 g, 0.5 mmol) and NaOH (0.040 g, 1 mmol) were dissolved in deionized water (7.5 mL) and stirred for 30 min at room temperature. In a separate beaker CuCl_2_·2H_2_O (0.085 g, 0.5 mmol) was dissolved in deionized water (5 mL) and AgNO_3_ powder (0.255 g, 1.5 mmol) was added. The AgCl precipitate which formed was filtered off and phen (0.099 g, 0.5 mmol) was added to the clear filtrate. This mixture was added into the solution of H_2_pydco and NaOH and stirred for 1 h at room temperature. The mixture was sealed in a 25 mL Teflon-lined reactor. The reactor was heated at 130 °C for 8 h and then cooled to room temperature at a rate of 10 °C h^−1^. Blue acicular crystals of 9 (m.p. 245 °C) were obtained and the final yield of 9 was 0.25 g (40% based on Cu). Anal. calcd for C_19_H_15_AgCuN_4_O_10_: C 36.18; H 2.40; N 8.88%. Found: C 36.15; H 2.18; N 8.93%. IR bands (KBr pellet, cm^−1^): 3416(br), 1743(w), 1649(s), 1552(w), 1515(w), 1382(s), 1209(m).

### Theoretical methods

The calculations of the noncovalent interactions were computed using the Gaussian-09 program package.^42^ We have used the M06-2X DFT functional in combination with Grimme's dispersion correction^43^ because it is convenient for describing the weak noncovalent interactions properly. Moreover, it is recommended for system with transition metals.^44^ In order to describe correctly the π–hole interactions we have used the crystallographic coordinates and the def2-TZVP basis set for all atoms. This procedure and level of theory have been successfully used to evaluate similar interactions.^45^ The interaction energies were computed by calculating the difference between the energies of isolated monomers and their assembly. The interaction energies were calculated with correction for the basis set superposition error (BSSE) by using the Boys–Bernardi counterpoise technique.^46^ The NCI plot^47^ iso-surfaces have been used to characterize noncovalent interactions. They correspond to both favorable and unfavorable interactions, as differentiated by the sign of the second density Hessian eigenvalue and defined by the isosurface color. The color scheme is a red-yellow-green-blue scale with red for *ρ*_cut_^+^ (repulsive) and blue for *ρ*_cut_^−^ (attractive).^48^ The Gaussian-09 M06-2X/def2TZVP level of theory wave function has been used to generate the NCI plot and the MEP surfaces.

### Potentiometric pH titrations

Potentiometric measurements were performed for solutions in a 50 mL double-walled glass vessel using a Model 686 Metrohm Tiroprocessor equipped with a combined glass-calomel electrode. The temperature was fixed at 25.0 ± 0.1 °C. The ionic strength was adjusted to 0.1 M by use of KNO_3_. A CO_2_-free atmosphere for the base (carbonate-free 0.095 M sodium hydroxide) was ensured throughout. The concentrations of H_2_pydco (L) and phen (Q) were 3.0 × 10^−3^ M and potentiometric pH titrations carried out in the absence and presence of 1.5 × 10^−3^ M of the metal ions. Metal complexes protonation and stability constants and ligands protonation constants were calculated using the program BEST introduced by Martell and Motekaitis.^49^ The value of KSH = [H^+^][OH^−^], for aqueous solution was 10^−13.781^.^50–53^

## Results and discussion

### Synthesis

The H_2_pydco ligand was synthesized by treating H_2_pydc with 30% H_2_O_2_ as oxidizing agent and Na_2_WO_4_·2H_2_O as catalyst, thereby converting the pyridine nitrogen atom to an *N*-oxide. H_2_pydc is a N/O donor ligand whereas H_2_pydco is exclusively an O-donor, a difference which dramatically affects both the molecular and supramolecular structures of the complexes it forms. Our oxidation method affords a 95% yield of pyridine-*N*-oxide-2,5-dicarboxylic acid. The synthesis of this ligand (H_2_pydco) was achieved by a modified extent of oxidizing agent (H_2_O_2_) from H_2_pydc.^54^

### Infrared spectroscopy

The broad and strong bands at 3000–3500 cm^−1^ can be attributed to the stretching vibrations *ν*(OH) of lattice and coordinated water molecules and *ν*(=C–H) in the aromatic rings. They are also indicative of the presence of hydrogen bonding.^13,55^ The strong *ν*_as_(COO^−^) and the *ν*_s_(COO^−^) bands in free H_2_pydco 1726 and 1419 cm^−1^ are shifted in the complexes to the lower frequencies in the range 1674–1628 cm^−1^ and 1398–1372 cm^−1^, respectively. These results indicate that in these complexes the carboxylate group of pydco coordinates to the transition metal ions through deprotonation.^56^ Furthermore, the strong absorption bands in the range of 1550–1600 cm^−1^ can be attributed to the *ν*(C=C) and *ν*(C=N) vibration of aromatic pyridyl ring for all these complexes. The IR spectrum of H_2_pydco (see Fig. S4†) shows a strong band at 1230 cm^−1^ due to the presence of N–O group confirming the synthesis of the ligand.^27^ Bands in the 1228–1205 cm^−1^ region for the complexes (see Fig. S4†) were therefore assigned to the N–O stretching vibration this group. The bands at 900–700 cm^−1^ are characteristic of the bending vibration *δ*(=C–H) in aromatic rings.

### NMR study

In the ^1^H NMR spectrum of H_2_pydco, the aromatic protons of the pyridyl ring are revealed as doublet of doublets at 8.36 ppm for the H_b_ proton, due to the vicinal couplings with both H_c_ and H_a_ protons (^3^*J*_H–H_ = 8.4 and ^4^*J*_H–H_ = 1.5 Hz). The H_c_ proton shows a doublet signal at 8.52 ppm with coupling to the H_b_ proton (^3^*J*_H–H_  =  8.1 Hz). The H_a_ proton appears as a doublet at 8.96 ppm, with coupling to the H_b_ proton (^4^*J*_H–H_ = 1.2 Hz) (see Fig. S2†). In the ^13^C{^1^H} NMR spectrum of H_2_pydco, the signals at 129.08, 131.55, 133.81, 138.60, and 139.88 ppm are related to the carbon atoms of the pyridyl ring of H_2_pydco (C2, C3, C4, C5, and C1), respectively. Both carbon atoms of the two carboxylic acid groups (C6 and C7) are revealed at 160.95 and 162.70 ppm, respectively (see Fig. S3†).

### CSD search

**Fig. 1:**
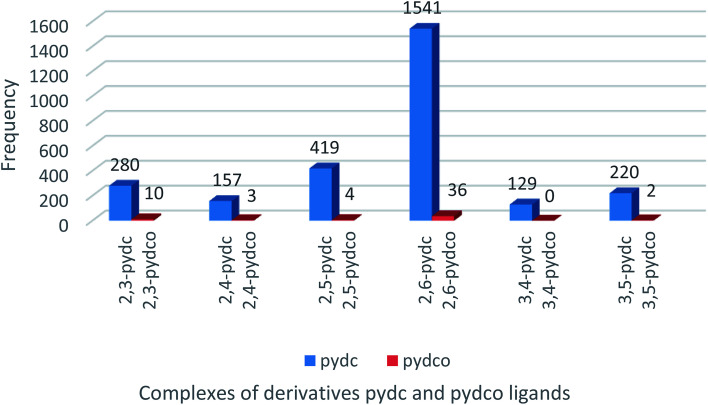
Frequency of complexes containing all isomers of the pydc and pydco ligands.

A search of the Cambridge Structural Database (CSD) for complexes of pyridine-2,5-dicarboxylic acid ligands (Fig. 1) returned 419 entries of complexes with 2,5-pydc, but only four complexes containing 2,5-pydco, the latter being lanthanoid complexes.^27^ This dearth of structurally-characterized complexes of all isomers of pydco spurred our efforts to prepare and characterize new supramolecular coordination complexes of 2,5-pydco with various transition metals.

### Description of the crystal structures

The crystallographic data for compounds 1–9 is shown in Table 1. In addition, selected bond lengths, valence angles and hydrogen bond geometries are given in Tables S1 and S2 in the ESI.†

Crystal data, data collection, and refinement parameters for 1–9

**Table:** 

	1	2	3	4	5
Empirical formula	C_14_H_26_Co_2_N_2_O_20_	C_7_H_11_CoNO_9_	C_7_H_11_NNiO_9_	C_7_H_11_NO_9_Zn	C_17_H_23_CoN_3_O_11_
Formula weight	660.23	312.10	311.86	318.56	504.31
*T* (K)	293(2)	298(2)	298(2)	298(2)	298(2)
Wavelength (Å)	0.71073	0.71073	0.71073	0.71073	0.71073
Crystal system	Monoclinic	Triclinic	Triclinic	Triclinic	Triclinic
Space group	*C*2/*c*	*P*1̄	*P*1̄	*P*1̄	*P*1̄
*a* (Å)	12.9792(19)	9.902(2)	9.858(2)	9.889(2)	9.3305(19)
*b* (Å)	9.746(3)	10.698(2)	10.656(2)	10.653(2)	9.882(2)
*c* (Å)	19.835(4)	11.742(2)	11.692(2)	11.751(2)	11.820(2)
*α* (°)	90	68.84(3)	68.85(3)	68.87(3)	83.25(3)
*β* (°)	105.290(14)	84.28(3)	84.18(3)	84.25(3)	78.22(3)
*γ* (°)	90	66.30(3)	66.00(3)	66.44(3)	89.16(3)
*V* (Å^3^)	2420.2(10)	1060.7(5)	1045.0(3)	1057.1(5)	1059.5(4)
*Z*	4	4	4	4	2
*D*_calc_ (g cm^−3^)	1.812	1.954	1.982	2.001	1.581
*μ* (mm^−1^)	1.469	1.663	1.902	2.369	0.875
*F*(000)	1352	636	640	648	522
Crystal size (mm^3^)	0.36 × 0.36 × 0.15	0.50 × 0.20 × 0.20	0.50 × 0.25 × 0.20	0.50 × 0.30 × 0.20	0.50 × 0.50 × 0.50
*θ* Range for data collection (°)	2.129 to 24.963	2.22 to 29.19	2.23 to 29.20	2.47 to 29.24	2.55 to 29.17
Index ranges	−15 ≤ *h* ≤ 14	−13 ≤ *h* ≤ 13	−13 ≤ *h* ≤ 13	−11 ≤ *h* ≤ 13	−10 ≤ *h* ≤ 12
	0 ≤ *k* ≤ 11	−14 ≤ *k* ≤ 14	−14 ≤ *k* ≤ 13	−14 ≤ *k* ≤ 14	−13 ≤ *k* ≤ 13
	0 ≤ *l* ≤ 11	−16 ≤ *l* ≤ 12	−16 ≤ *l* ≤ 14	−16 ≤ *l* ≤ 15	−16 ≤ *l* ≤ 16
Reflections collected	1471	11 589	11 560	11 619	11 927
Independent reflections	1403 [*R*_int_ = 0.0331]	5672 [*R*_int_ = 0.0505]	5590 [*R*_int_ = 0.0409]	5656 [*R*_int_ = 0.1040]	5672 [*R*_int_ = 0.0545]
Data/restraints/parameters	1403/9/226	5672/2/371	5590/0/325	5656/6/373	5672/10/325
GOF on *F*^2^	1.037	0.892	1.000	1.028	1.083
Final *R* indices [*I* > 2*σ*(*I*)]	*R*_1_ = 0.0423 w*R*_2_ = 0.1067	*R*_1_ = 0.0324 w*R*_2_ = 0.0668	*R*_1_ = 0.0283 w*R*_2_ = 0.0710	*R*_1_ = 0.0528 w*R*_2_ = 0.1217	*R*_1_ = 0.0428 w*R*_2_ = 0.1107
*R* Indices (all data)	*R*_1_ = 0.0591 w*R*_2_ = 0.1154	*R*_1_ = 0.0574 w*R*_2_ = 0.0716	*R*_1_ = 0.0412 w*R*_2_ = 0.0742	*R*_1_ = 0.0763 w*R*_2_ = 0.1339	*R*_1_ = 0.0537 w*R*_2_ = 0.1162
Largest diff. peak and hole (e Å^−3^)	0.597 and −0.677	0.432 and −0.376	0.415 and −0.549	1.030 and −1.315	0.462 and −0.424

**Table:** 

	6	7	8	9
Empirical formula	C_19.36_H_26.35_CoN_3_O_12.32_	C_17_H_16_ClCuN_3_O_7_	C_34_H_27_ClCu_2_N_6_O_12_	C_19_H_15_AgCuN_4_O_10_
Formula weight	556.86	473.33	874.17	630.76
*T* (K)	120(2)	298(2)	298(2)	298(2)
Wavelength (Å)	0.71073	0.71073	0.71073	0.71073
Crystal system	Triclinic	Triclinic	Triclinic	Triclinic
Space group	*P*1̄	*P*1̄	*P*1̄	*P*1̄
*a* (Å)	7.9216(16)	8.5223(17)	8.7022(17)	9.0136(18)
*b* (Å)	9.862(2)	9.4697(19)	9.913(2)	10.366(2)
*c* (Å)	16.736(3)	12.214(2)	10.553(2)	12.609(3)
*α* (°)	91.96(3)	75.25(3)	98.76(3)	113.63(3)
*β* (°)	102.07(3)	84.25(3)	90.30(3)	91.47(3)
*γ* (°)	108.51(3)	80.60(3)	108.64(3)	106.35(3)
*V* (Å^3^)	1205.3(5)	938.7(3)	851.1(3)	1022.8(5)
*Z*	2	2	1	2
*D*_calc_ (g cm^−3^)	1.535	1.675	1.706	2.048
*μ* (mm^−1^)	0.781	1.353	1.404	2.067
*F*(000)	578	482	444	626
Crystal size (mm^3^)	0.50 × 0.50 × 0.45	0.30 × 0.10 × 0.05	0.45 × 0.25 × 0.20	0.5 × 0.25 × 0.25
*θ* Range for data collection (°)	2.504 to 29.177	2.43 to 29.16	2.20 to 29.14	2.21 to 29.19
Index ranges	−10 ≤ *h* ≤ 10	−11 ≤ *h* ≤ 11	−11 ≤ *h* ≤ 11	−12 ≤ *h* ≤ 12
	−11 ≤ *k* ≤ 13	−12 ≤ *k* ≤ 12	−13 ≤ *k* ≤ 12	−14 ≤ *k* ≤ 14
	−22 ≤ *l* ≤ 22	−12 ≤ *l* ≤ 16	−14 ≤ *l* ≤ 14	−17 ≤ *l* ≤ 16
Reflections collected	13 150	10 450	9424	11 268
Independent reflections	6451 [*R*_int_ = 0.0606]	5021 [*R*_int_ = 0.1092]	4556 [*R*_int_ = 0.0554]	5486 [*R*_int_ = 0.0504]
Data/restraints/parameters	6451/29/396	5021/2/277	4556/2/256	5486/6/328
GOF on *F*^2^	1.041	0.966	0.977	0.988
Final *R* indices [*I* > 2*σ* (*I*)]	*R*_1_ = 0.0478 w*R*_2_ = 0.1120	*R*_1_ = 0.0676 w*R*_2_ = 0.1196	*R*_1_ = 0.0411 w*R*_2_ = 0.0911	*R*_1_ = 0.0499 w*R*_2_ = 0.1291
*R* Indices (all data)	*R*_1_ = 0.0619 w*R*_2_ = 0.1200	*R*_1_ = 0.1329 w*R*_2_ = 0.1388	*R*_1_ = 0.0622 w*R*_2_ = 0.0975	*R*_1_ = 0.0763 w*R*_2_ = 0.1406
Largest diff. peak and hole (e Å^−3^)	0.66 and −0.86	0.791 and −0.517	0.545 and −0.405	1.377 and −0.931

### Crystal structure of 1

**Fig. 2:**
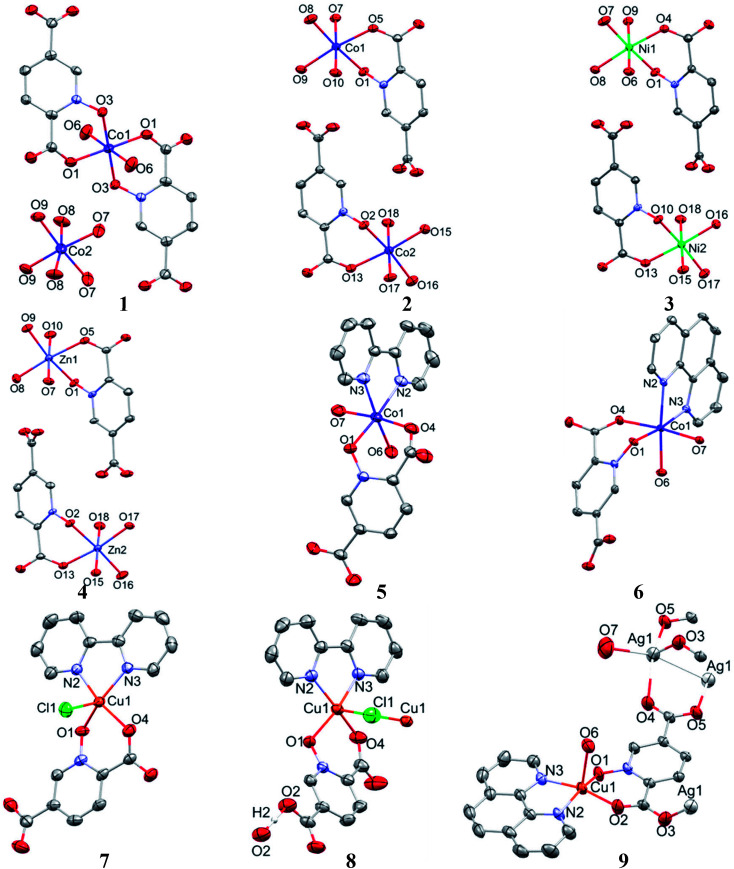
Molecular structures for complexes 1–9, with selected atoms labeled and displacement ellipsoids at 50% probability. For the sake of clarity, NO_3_^−^ counterion, solvent molecules and H atoms are omitted.

**Fig. 3:**
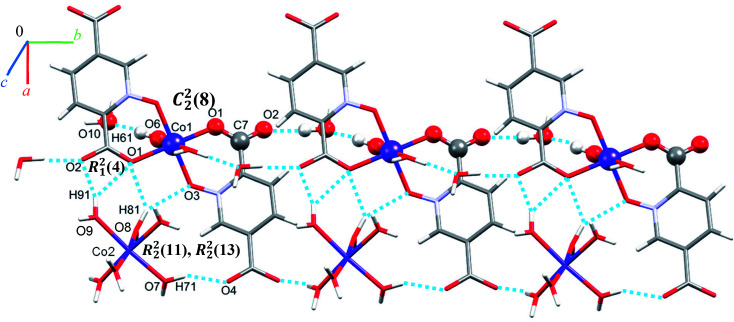
Representation of the linear chain along the *b* axis in 1.

**Fig. 4:**
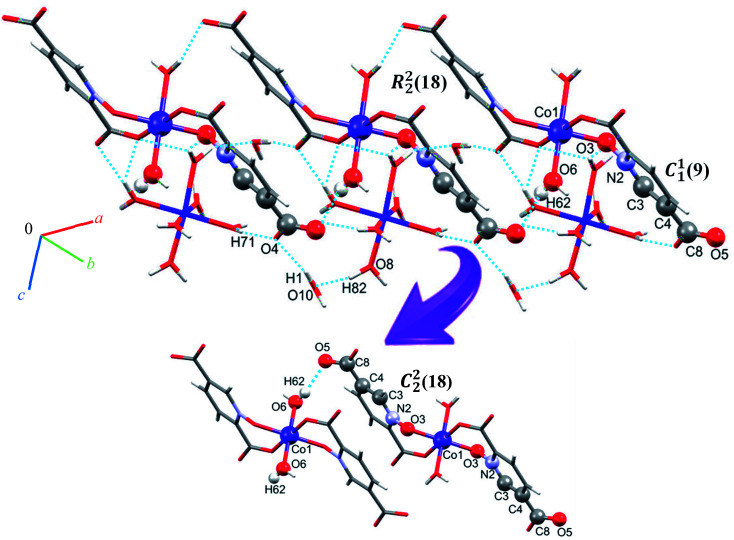
Extended one-dimensional structure of 1 along the side view formed by hydrogen bonding described by graph-set notations *R*^2^_2_(18), *C*^1^_1_(9) and *C*^2^_2_(18).

Single crystal X-ray diffraction analysis reveals that 1 crystallizes in the monoclinic space group *C*2/*c*. The asymmetric unit of 1 contains two Co(ii) ion, one pydco^2−^ anion, four coordinated water molecules (one and three for Co1 and Co2, respectively) and one uncoordinated water molecule (see Fig. 2). Co1 is coordinated by two mutually *trans* carboxylate O donors and two mutually *trans N*-oxide O donors from two pydco^2−^ ligands to form the equatorial plane, and by two apical oxygen atoms from two water molecules to complete the distorted octahedral geometry. Co2 is coordinated by six water molecules in a regular octahedral geometry. The O–H⋯O hydrogen bonds create motifs of graph-set notation *R*^2^_1_(4), *R*^2^_2_(11), *R*^2^_2_(13) and *C*^2^_2_(8) which extend complex 1 along the crystallographic *b* direction. Interestingly, the structure exhibits bifurcated O–H⋯O interactions with the oxygen atom of the 5-carboxylate group acting as a hydrogen bond acceptor from two different O–H donors, while two of the water molecules coordinated to Co2 act as bifurcated donors (see Fig. 3). Each unit comprising one cationic and one anionic complex is linked together through O–H⋯O hydrogen bonds between uncoordinated water molecules and 2-carboxylate oxygen atoms or water molecules coordinated to Co2, resulting in a zigzag chain structure along the *c* direction, as shown in Fig S5.† In addition, there are strong O–H⋯O hydrogen bonds in another chain which form motifs with the graph-set notations *R*^2^_2_(18), *C*^1^_1_(9) and *C*^2^_2_(18) (see Fig. 4). Consequently, the three-dimensional network in 1 made by all above intermolecular O–H⋯O and C–H⋯O interactions.

### Crystal structures of 2, 3, and 4

**Fig. 5:**
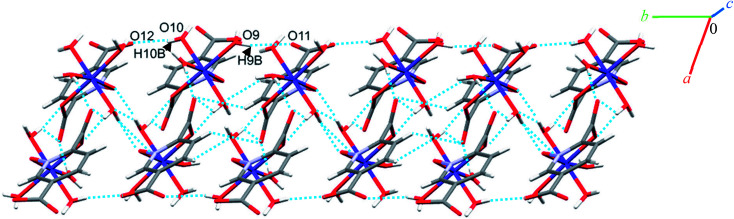
Representation of the one-dimensional linear chain along the *b* axis in 2.

Complexes 2, 3 and 4 are isomorphous and isostructural, crystallizing in the triclinic space group *P*1̄. The asymmetric unit of each complex contains two independent [M(pydco)(H_2_O)_4_] units (M = Co, Ni, and Zn), each consisting of one metal ion on a general position and in a distorted octahedral environment, with one bidentate pydco^2−^ ligand and two water molecules occupying the equatorial plane, while the axial positions are occupied by two water molecules (see Fig. 2). Taking 2 as representative of the three isostructural complexes, we find that it forms dimers *via* O–H⋯O hydrogen bonds producing motifs with graph-set notations *R*^2^_2_(8) and *R*^2^_2_(18). In addition, we identified weak C–O⋯π interactions (with a centroid⋯O12 separation of 3.732 Å).^16,57^ These dimers form chains along the *c* direction *via* O15–H15A⋯O9 hydrogen bonds (H15A⋯O9 = 2.235 Å) between water molecules coordinated to Co^II^ (see Fig. S6†). O–H⋯O and N–H⋯O hydrogen bonds generate chains along the *b* axis, forming motifs with graph-set notation *R*^2^_2_(8), *C*^2^_2_(13) and *C*^2^_2_(15) (see Fig. 5 and S7†).

Additionally, weak C–H⋯π interactions (with a centroid⋯H12C12 separation of 3.334 Å)^58^ link dimers into chains along the *a* axis, thereby completing a three-dimensional network (see Fig. S8†). Additional interactions are analyzed below in the theoretical study.

### Crystal structure of 5

Complex 5 crystallizes in the triclinic space group *P*1̄. The Co(ii) ion (see Fig. 2) occupies a general position and exhibits distorted octahedral coordination from one bidentate pydco^2−^ ligand, one bipy ligand and two water molecules. The equatorial plane is occupied by one water molecule, two bipy nitrogen atoms and O1 from an *N*-oxide, while axial positions are occupied by one oxygen atom (O4) from position 2 of carboxyl group and another water molecule.

**Fig. 6:**
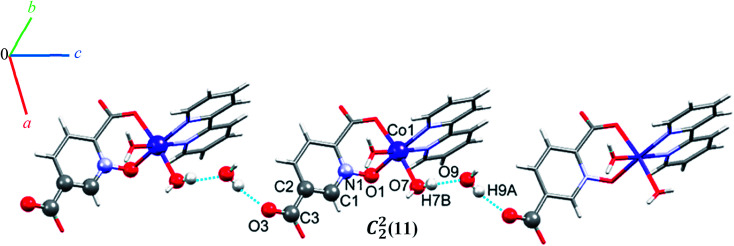
Representation of the one-dimensional chain along the *c* axis in 5.

O–H⋯O hydrogen bonding interactions between water molecules, two carboxylate groups oxygen atoms and an *N*-oxide from a pydco ligand result in an extended chain along the *b* direction *via* motifs with graph-set notations *R*^2^_2_(8), *R*^2^_2_(18) and *R*^4^_4_(16). Weak C–O⋯π interactions (with a centroid⋯O2C3 separation of 3.716 Å) reinforce this chain structure (see Fig. S9†). A *C*^2^_2_(11) motif is generated along the *c* axis by hydrogen bonding interactions (H7B⋯O9: 1.93(2) Å and H9A⋯O3: 1.94(4) Å) between water molecules and oxygen acceptors of the 5-carboxylate group (Fig. 6).

In addition, hydrogen bonding (C11H11⋯O4; 2.440 Å) and two types of π⋯π stacking between pyridine rings (with two different types of centroid⋯centroid separations of 3.585, and 3.606 Å) leads to a third chain along the *a* axis (see Fig. S10†).^59^ In general, the chains are packed *via* O–H⋯O, C–O⋯π, π⋯π stacking interactions in three directions to cooperate three-dimensional supramolecular structure.

### Crystal structure of 6

A view of complex 6 with labeling of selected atoms is shown in Fig. 2. The asymmetric unit contains a Co(ii) ion occupying a general position and exhibiting distorted octahedral coordination geometry, in which the apical positions are occupied by O6 from a water molecule and N2 from a phen ligand; the equatorial plane is defined by O7 from water molecule, N3 from a phen ligand, O1 from an *N*-oxide group and O4 from the 2-carboxylate group of a pydco^2−^ ligand.

**Fig. 7:**
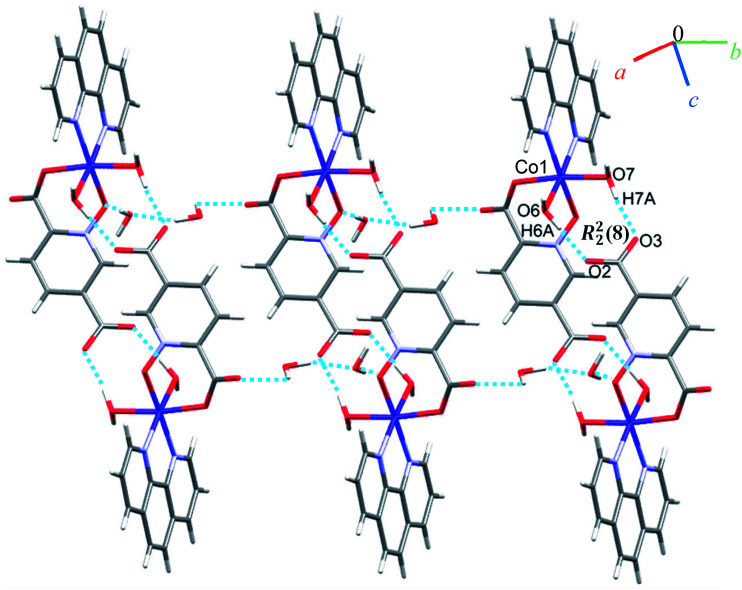
Representation of the chain along the *b* axis in 6.

**Fig. 8:**
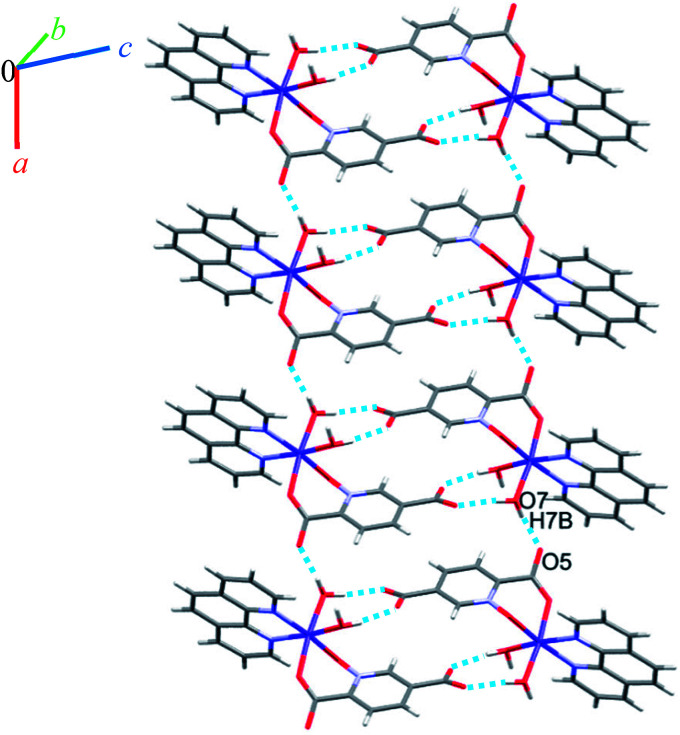
View of the ladder running parallel to the *a* axis in 6.

Due to the presence of disorder, it was a challenge to identify the solvent molecules, but we have been able to conclude that the asymmetric unit contains five molecules of water and 0.2 molecules of ethanol. Molecules of 6 are linked into dimers by O–H⋯O hydrogen bonds between two water molecules from one complex and two oxygen atoms of the 5-carboxylate group from an adjacent complex, resulting in homosynthons with graph-set notation *R*^2^_2_(8). Dimers are linked into chains along the *b* axis by two lattice water molecules which form O–H⋯O hydrogen bonds between oxygen atoms from an *N*-oxide group and from an O atom from the 2-carboxylate group (see Fig. 7). The dimers repeat along *a* axis *via* O–H⋯O hydrogen bonds between a coordinated water molecule and oxygen atoms of 2-carboxylate group from pydco^2−^ ligand forming one-dimensional ladders (see Fig. 8). These ladders are attached to each other *via* hydrogen bonding (C17H17⋯O4; 2.510 Å), π⋯π (with two different types of centroid⋯centroid separations of 3.627 and 3.671 Å), CH⋯π (with a centroid⋯H18C18 separation of 3.5325 Å), and C–O⋯π (with a centroid⋯O5C7 separation of 3.317 Å) interactions between pyridine and phenyl rings of phen and thereby generate two-dimensional sheets (Fig. S11 and S12†).

### Crystal structure of 7

**Fig. 9:**
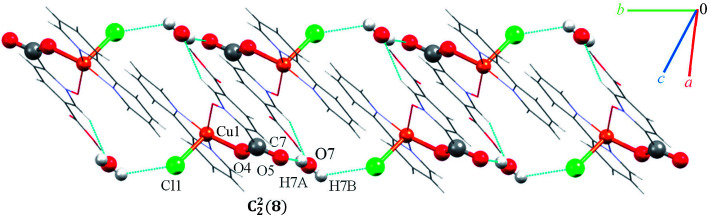
Representation of the chain running parallel to the *b* axis in 7.

Complex 7 crystallizes in the triclinic space group *P*1̄. The asymmetric unit contains a five-coordinated complex of Cu(ii) which adopts a distorted square pyramidal coordination geometry‡‡The distorted square pyramidal coordination geometry is indicated by the value of *τ*_5_ (0.230). The parameter *τ*_5_ is defined as (*β* − *α*)/60, where *β* and *α* are the largest angles subtended at the metal center). For ideal square pyramidal *τ*_5_ is 0 and for ideal trigonal bipyramidal geometry is 1.^60^ in which the basal plane is occupied by two bipy nitrogen atoms and two oxygen atoms from one Hpydco^−^ ligand, while the axial position is occupied by a chloride ion. The asymmetric unit is completed by two uncoordinated water molecules. A displacement ellipsoid plot of complex 7 with selected atoms labeling scheme is shown in Fig. 2. The chloride anions and uncoordinated water molecules participate in O–H⋯O and O–H⋯Cl hydrogen bonding and thereby form chains along the *b* axis with motifs described by the graph-set notation *C*^2^_2_(8) (see Fig. 9).

Another chain is generated through weak C–H⋯O, C–H⋯Cl, C–H⋯π (with a centroid⋯H5C5 separation of 3.535 Å), and π⋯π (with a centroid⋯centroid separations of 3.631 Å) interactions (see Fig. S13 in the ESI†). Moreover, the third dimension is generated *via* strong O–H⋯O hydrogen bonds between water molecules and 5-carboxylate groups of pydco^2−^ which lead to the formation of a motif described by the graph-set notation *R*^4^_4_(12) (see Fig. S14 in the ESI†). According to Fig. S15 in the ESI,† the three-dimensional structure of compound 7 has expanded *via* different interactions by connection of all above chains.

### Crystal structure of 8

Single crystal X-ray diffraction analysis shows that compound 8 crystallizes in the triclinic space group *P*1̄. A displacement ellipsoid drawing of 8 with selected atoms labelled is shown in Fig. 2. The asymmetric unit of 8 contains one Cu(ii) ion, one bipy ligand, one Hpydco^−^ anion, one chloride anion and one uncoordinated water molecule wherein hydroxyl proton H2 is shared between two O2 atoms with 50% occupancy.^61^ A distorted square pyramidal geometry, is defined by two nitrogen atoms from a bipy ligand and two oxygen atoms from a Hpydco^−^ ligand forming the equatorial plane with a chloride anion in the axial position.

**Fig. 10:**
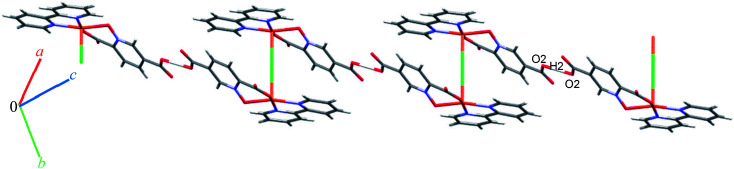
Representation of the zigzag chain along the side view in 8.

Molecules form zigzag chains *via* alternating chloride bridges between Cu(ii) ions and O2H2⋯O2 hydrogen bonds (see Fig. 10).

(H6B⋯O6; 2.59(3) Å) and (C1H1⋯O6; 2.554 Å) hydrogen bonds between water molecules and C1 of pyridine ring from pydco^2−^ ligand cross-ink the zigzag chains into two-dimensional sheets. These O–H⋯O hydrogen bonds lead to the formation of a motif with the graph-set notation *R*^2^_2_(4). Weak π⋯π interactions with a centroid⋯centroid separations of 3.775 Å occur between the pyridine rings of two bipy ligands in adjacent chains (see Fig. S16†). The structure is extended parallel to a axis *via* weak C–H⋯O interactions between uncoordinated water molecules and the pyridine rings of pydco^2−^ and bipy ligands (see Fig. S17†). Finally, covalent bonds and non-covalent interactions such as hydrogen bonds and π⋯π stacking in these three chains expand the structure to give a three-dimensional network (Fig. S18†).

### Crystal structure of 9

Complex 9 is a coordination polymer that crystallizes in the triclinic space group *P*1̄. The structure consists of a cationic complex [AgCu(H_2_O)_2_(phen)(pydco)]_*n*_^+^ with a nitrate as counterion (see Fig. 2). The asymmetric unit contains one Cu(ii) and one Ag(i) cation, one pydco^2−^ and one phen ligand and the aforementioned nitrate anion. Cu(ii) (*τ*_5_ = 0.105) is five-coordinate with distorted square pyramidal geometry. For the Cu(ii) center, the basal plane is defined by two oxygen atoms (O1 and O2) from one pydco^2−^ ligand and two nitrogen atoms (N2 and N3) from a phen ligand, while the axial position is occupied by one oxygen atom (O6) of a coordinated water molecule. The Ag(i) complex forms an acetate-bridged dimer with an Ag⋯Ag separation of 2.886(1) Å. The coordination around the Ag(i) metal ion is four-coordinate (by O3, O7, O4 and O50) and its geometry is distorted tetrahedral. In 9, the pydco^2−^ ligand is completely deprotonated and coordinated to metal ions as a pentadentate ligand. It is worth noting that pydco^2−^ chelates to the Cu(ii) ion through the oxygen atoms of the *N*-oxide (O1) and carboxylate (O2) while another oxygen of this carboxylate (O3) is coordinated to Ag. The oxygen atoms of the 5-carboxyl group coordinates in a bidentate fashion to Ag1. In this structure the free nitrate anion has a degree of disorder, perhaps because the data were acquired at room temperature, and no suitable modelling of this disorder could be achieved.

Monomer units form ladder parallel to the *b* axis *via* coordinate bonds (Ag1–O3) and two types of interactions increase the stability of these monomers: the first is O–H⋯O hydrogen bonding interactions between coordinated water molecules and nitrate ions and the second type is π⋯π stacking interactions between two pyridine rings of pydco^2−^ ligands (with a centroid⋯centroid separation of 3.639 Å).

**Fig. 11:**
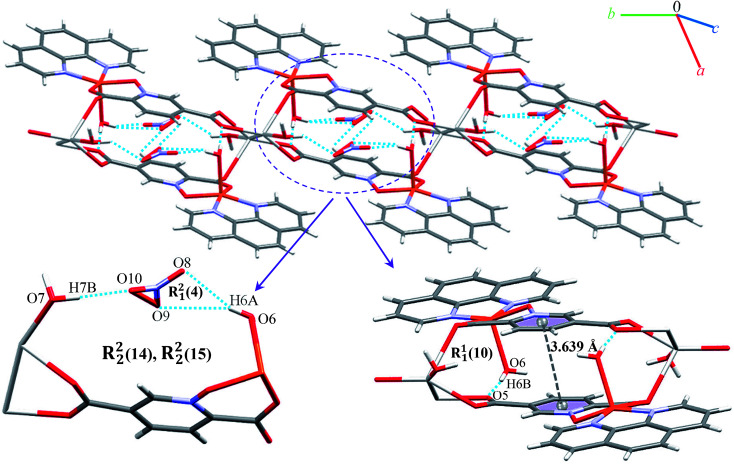
Structural representations and graph-set notation for the ladder parallel to the *b* axis assembled *via* hydrogen bonding and π⋯π interactions in 9. The hydrogen bonds and π⋯π interactions are shown as blue dotted lines and gray dashed lines, respectively.

**Fig. 12:**
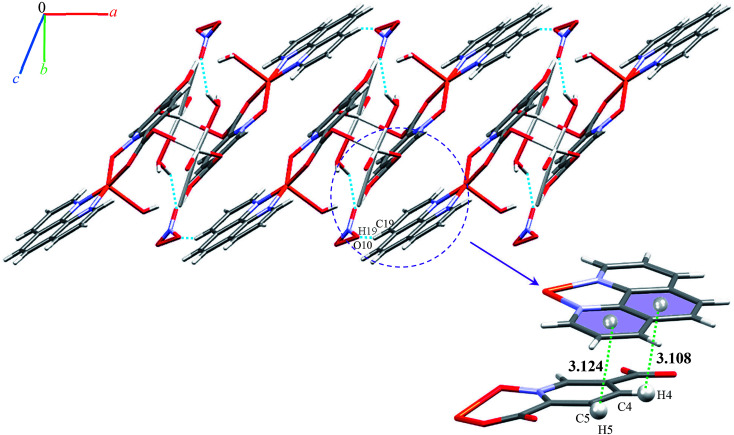
Representation of the chain in compound 9, assembled *via* O–H⋯O and C–H⋯O hydrogen bonding and C–H⋯π interactions, running parallel to the *a* axis. Distances are given in Å. The hydrogen bonds and C–H⋯π interactions are shown as blue and green dotted lines, respectively.

We identified cyclic synthons with graph-set notations *R*^2^_1_(4), *R*^1^_1_(10), *R*^2^_2_(14) and *R*^2^_2_(4) (see Fig. 11). Another one-dimensional chain was formed *via* weak interactions C13H13⋯O8 and C4H4⋯O9 between phenyl ring of phen, pyridine ring of pydco^2−^ ligands and nitrate anion as well as weak π⋯π interactions between phenyl rings of two phen ligands (with a centroid⋯centroid separations of 3.535 Å) and C–H⋯π interactions (with a centroid⋯H13C13 separations of 3.617 Å) (see Fig. S19†). Molecules are linked into chains running parallel to the *a* axis *via* different types of interactions including O7–H7A⋯O8, C19H19⋯O10, C–H⋯π interactions (with centroid⋯H4C4 and centroid⋯H5C5 separations of 3.108 and 30 124 Å, respectively) (see Fig. 12).

Thus, a one-dimensional coordination polymer is linked by further interactions into a three-dimensional network (see Fig. S20†).

### Comparisons

**Fig. 13:**
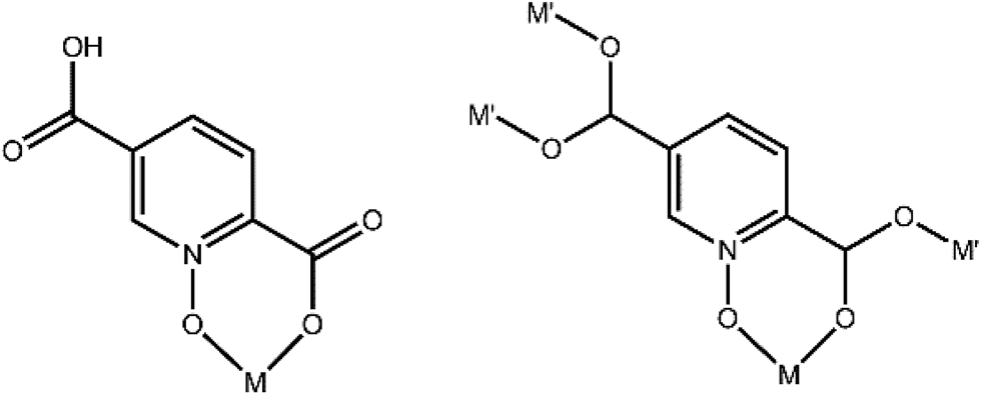
Hpydco^−^ and pydco^2−^ coordination modes observed in 1–9.

A search of the CSD (version 5.38, with updates until February 2017) for the pydco ligand returned no published examples of transition metal complexes coordinated to this ligand. Previously in our group we have synthesized a series of complexes with N/O donor ligand H_2_pydc^62–65^ and here we report the corresponding transition metal complexes with exclusively O-donor H_2_pydco ligand. The aim was to explore how altering the coordination mode of the ligand changes the molecular structures, non-covalent interactions and therefore the supramolecular structures of these complexes. The first-row transition metals are borderline acids and they have a greater affinity for nitrogen than for oxygen atoms. However, it could be anticipated that the presence of two donor oxygen atoms in the H_2_pydco ligand would lead to their chelating the metal ion and the formation of a six-membered ring. Indeed, if the ligand can assume a suitable conformation, the formation of six-membered ring causes little or no strain on the bond angles of the ligand or at the metal.^66^ We observed two coordination modes for the pydco ligand in our complexes (see Fig. 13).

Based on the CSD search, there are three complexes with the same structures as complexes 1, 3, and 6 in this work. In Table 2, M–O_*N*-oxide_, M–O_coo_ and M–N_pyridine_ distances are shown. Since complexes 2, 3, and 4 are isomorphous, a comparison was performed between corresponding M–O_*N*-oxide_ and M–O_COO_ distances against the relevant ionic radii for Co(ii), Ni(ii), and Zn(ii) (Table 2). Average bond distances for these three complexes show that M–O_mean*N*-oxide_ is longer than M–O_meanCOO_.

**Table:** Comparison of the bond distance (Å) for 1–9 and some pydc^2−^ complexes

Complexes	M–N_*N*-oxide_	M–O_COO_	M–N_pyridin_	Ref.
1	2.018(3)	2.032(4)	—	This work
2	2.0991(17)	2.0901(17)	—	This work
3	2.0679(14)	2.0507(14)	—	This work
4	2.105(3)	2.141(3)	—	This work
5	2.101(2)	2.083(2)	—	This work
6	2.095(2)	2.059(2)	—	This work
7	1.957(4)	1.922(3)	—	This work
8	1.941(2)	1.927(2)	—	This work
9	1.932(3)	1.907(4)	—	This work
[Co(H_2_O)_6_][Co(pydc)_2_(H_2_O)_2_]·4H_2_O	—	2.075	2.136	67
[Co(H_2_O)_2_(phen)(pydc)]·H_2_O	—	2.064(2)	2.159(2)	68
[Ni(pydc)(H_2_O)_4_]·2H_2_O	—	2.048(1)	2.070(1)	69

The Cremer and Pople ring puckering parameters^70,71^ shown in Table 3 indicate that the six-membered chelating rings created by coordination of ligand to the metal ions adopt the following conformations:

**Table:** Cremer and Pople ring puckering parameters

	1	2	3	4	5	6	7	8	9
*q*_2_ (Å)	0.529(4), 0.529(4)	0.7070(19), 0.7239(19)	0.6996(17), 0.7033(17)	0.733(3), 0.735(3)	0.7629(17)	0.7126(19)	0.397(4)	0.571(2)	0.268(4)
*q*_3_ (Å)	−0.187(4), 0.187(4)	−0.1569(19), 0.1171(19)	−0.1582(17), 0.1288(17)	−0.122(3), 0.152(3)	0.2048(17)	−0.2127(19)	−0.137(4)	−0.248(2)	0.102(4)
*Φ*_2_ (°)	143.7(5), 323.7(5)	221.42(15), 46.12(15)	220.86(14), 44.57(14)	225.9(3), 42.2(3)	37.01(13)	215.20(15)	214.3(6)	204.8(2)	40.4(9)
*Θ*_2_ (°)	109.5(4), 70.5(4)	102.52(15), 80.81(15)	107.75(14), 79.63(14)	99.4(2), 78.4(2)	74.97(12)	106.62(15)	109.0(5)	113.51(18)	69.1(8)
*Q* (Å)	0.561(4), 0.561(4)	0.7239(17), 0.7330(18)	0.7170(16), 0.7154(16)	0.748(3), 0.751(3)	0.7896(16)	0.7437(17)	0.420(3)	0.6219(18)	0.287(4)

**Fig. 14:**
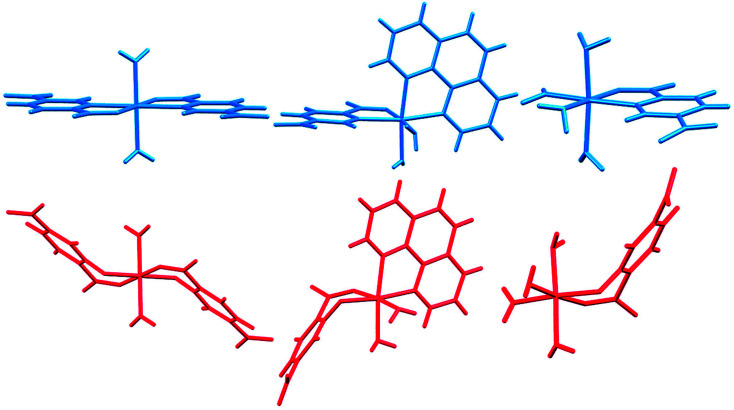
Comparison of the structures of 1, 3, and 6 (red) with complexes of the H_2_pydc ligand (blue).

(i) 1 has two rings with half-chair and skew–boat conformations;

(ii) Each of 2, 3 and 4 complexes have two rings in which they have a skew–boat conformation in one ring. In another ring, they have state between skew–boat and boat conformations.

(iii) 5 and 6 each have one ring with a skew–boat conformation;

(iv) 7, 8, and 9 each have one ring with a half-chair conformation;

In Fig. 14 we compare the structures of compounds 1, 3, and 6 with previously synthesized complexes of H_2_pydc.^67–69^ The formation of 5-membered chelate rings by the H_2_pydc ligand frequently resulted in complexes with planar conformations, while the H_2_pydco ligand formed complexes with 6-membered chelate rings displaying twisted conformations. *N*-oxide functionalization of the pyridine ring of the ligand led to the formation of complex and interesting supramolecular frameworks.

### Theoretical study

The theoretical study is devoted to analyze some unconventional non-covalent interactions observed in the solid-state structures of compounds 2–4. In particular, we have estimated the energy associated to π–hole interactions that are established between the coordinated carboxylate group and the uncoordinated one, which is the π–hole donor. It should be mentioned that π–hole interactions in X-ray structures have been studied by Bürgi and Dunitz in 1975,^72^ thus revealing the trajectory along which a nucleophile attacks the π–hole of carbonyl group. More recently, the importance of n → π* interactions in proteins from a lone pair of electrons (n) to the antibonding orbital (π*) of carbonyl group has been demonstrated.^73^ Moreover, operative π-holes have been described nitroderivatives,^74^ group 13 molecules^75^ and acyl carbon containing molecules.^76^

**Fig. 15:**
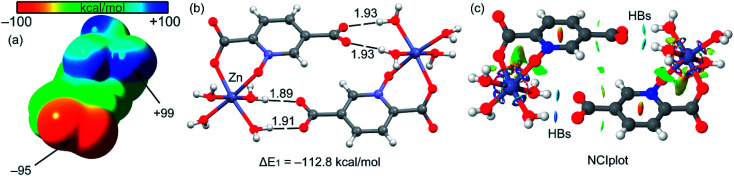
(a) MEP surface of compound 4. (b) H-bonded dimer of 4. Distances in Å. (c) NCI plot of the H-bonded dimer of compound 4. The gradient cut-off is *s* = 0.35 au, and the color scale is −0.04 < *ρ* < 0.04 au.

First of all, we have computed the molecular electrostatic potential (MEP) plotted onto the approximate van der Waals surface (isosurface 0.001 au) in order to investigate the electron rich and electron poor region of the complex. Since compounds 2–4 are isostructural, we have used compound 4 as a model because the closed-shell electronic configuration of this complex (d^10^ metal center) facilitates the computational analysis. The MEP surface of compound 4 is given in Fig. 14a and it can be observed the most positive region is located at the H-atoms of the coordinated water molecules (+99 kcal mol^−1^). This is due to the coordination of the water molecules to the metal center that increases the acidity of the H atoms. The most negative region is located at the O-atoms of the uncoordinated carboxylate group (−95 kcal mol^−1^), as expected. Therefore, the most favored interaction from an electrostatic point of view is an H-bond between the M − OH_2_ and the carboxylate group. As a matter of fact, this molecule forms self-assembled dimer in the solid state due to the formation of four strong H-bonds, as shown in Fig. 15b. The formation energy of this dimer is very large Δ*E*_1_ = −112.8 kcal mol^−1^, in good agreement with the MEP analysis and solid-state structure. In order to further characterize this assembly, we have used the NCI plot index computational tool. Non-covalent interactions are efficiently visualized and identified by using the NCI plot tool. It allows an easy assessment of host–guest complementarity and the extent to which weak interactions stabilize a complex. For the theoretical model of the assembly used in Fig. 15b we have computed the NCI plot that is represented in Fig. 15c. It can be observed several small and dark blue isosurfaces that characterize the intramolecular H-bonds that confirm the strong nature of these bonds.

**Fig. 16:**
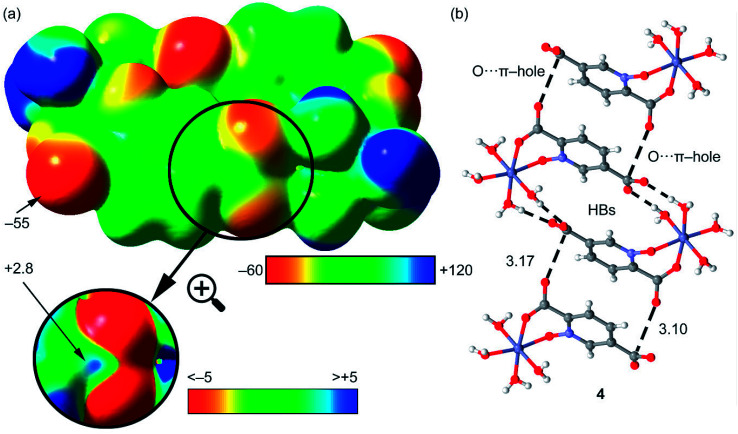
(a) MEP surface of the dimer of compound 4. The MEP values at selected points of the surface are indicated in kcal mol^−1^ (b) partial view of the X-ray structure of compound 4. Distances in Å.

It is important to highlight that the MEP surface of the monomer of compound 4 does not exhibits a positive π–hole over the carboxylate group, which is expected taking into consideration that the negative charge is located in this group. However, if the MEP surface is computed for the H-bonded dimer shown Fig. 15b, a small π–hole appears over the C atoms (see Fig. 16). The MEP surface shown in Fig. 16a also reveals a negative MEP at the O-atom of the coordinated carboxylate group (−55 kcal mol^−1^). The MEP over the C-atom of the carboxylate that establishes the double H-bond with the coordinated water molecules is very small +2.5 kcal mol^−1^ (see Fig. 16b). Nevertheless, it explains the formation of the infinite supramolecular chain in the solid state (see Fig. 16b) where the H-bonded self-assembled dimer interacts with the neighbor molecules in the X-ray structure by means of double π–hole interactions. Both O⋯C distances are shorter than the sum of their van der Waals radii (3.22 Å) and quite directional.

**Fig. 17:**
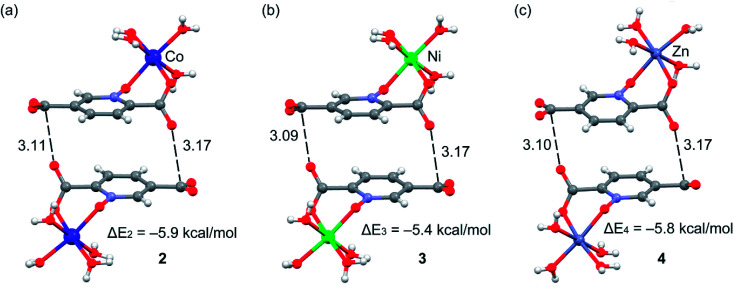
Formation energies of the π–hole dimers in compounds 2–4. Distances in Å.

We have evaluated energetically the π–hole complexes in complexes 2–4, see Fig. 17. It can be observed that the geometric features of the three complexes are almost identical and also the interaction energies, thus suggesting that the type of metal center has a little influence on the interaction energy. The interaction energies are weak (around 2.5 kcal mol^−1^ each O⋯π–hole) in good agreement with the small MEP value at the π–hole.

**Fig. 18:**
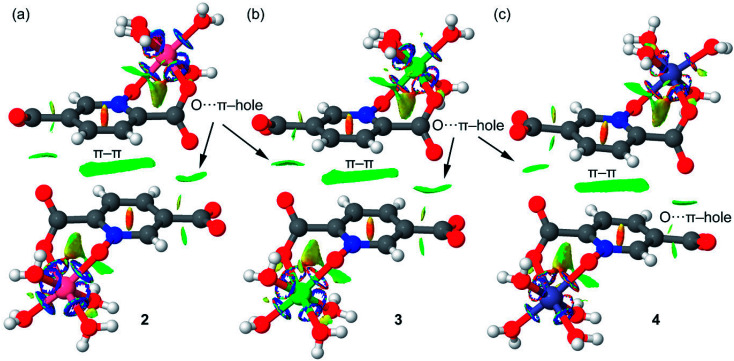
NCI plots of the π–hole dimers of compounds 2 (a), 3 (b) and 4 (c). The gradient cut-off is *s* = 0.35 au, and the color scale is −0.04 < *ρ* < 0.04 au.

We have also computed the NCI plots of the π–hole dimers represented in Fig. 18. The existence and weak attractive nature of the O⋯π–hole interactions is confirmed by the presence of green isosurfaces located between the O and C-atoms of carboxylate. Moreover, the NCI plot also reveals the existence of π–π stacking interactions since a more extended isosurface located between the aromatic ligands also appears upon complexation.

### Solution studies

In these experiments, the completely protonated forms of 2,5-pydco (L), 2,2′-bipyridine (Q) and 1,10-phenanthroline (Q′) were titrated with a standard NaOH solution in order to investigate their stoichiometry and protonation constants. The resulting values for the overall stability and stepwise protonation constants of L, Q and Q′ as well as the recognition constants for the L–Q and L–Q′ proton transfer systems are listed in Table 4. The resulting protonation constants are in satisfactory agreement with those reported in the literature; the observed difference is due to different conditions. The corresponding experimental pH titration profiles are shown in Fig. S21(a–c).† As can be seen, in all cases the potentiometric titration curves are depressed in the presence of the metal ions, indicating their strong interactions with metal ions. As it is obvious from Table 4, the most abundant proton transfer species for 2,5-pydco/2,2′-bipy system present at pH 2.3–2.6 (46.2%), and 3.7 (33.2%) are 2,5-pydcoH3—2,2′-bipy (log *K* = 7.72) and 2,5-pydcoH2—2,2′-bipy (log *K* = 3.93). In the 2,5-pydco/1,10-phen system the most abundant proton transfer species are 2,5-pydcoH2—1,10-phen (52.3%, log *K* = 10.47) and 2,5-pydcoH3—1,10-phen (34.0%, log *K* = 13.23).

**Table:** Overall stability and stepwise protonation constants of 2,5-pydco, 1,10-phenanthroline and 2,2′-bipyridine and recognition constants for their interaction in aqueous solution at 25 °C *μ* = 0.1 M of KNO_3_

Stoichiometry				log *β*	Equilibrium quotient *K*	log *K*	Max%	At pH
2,5-pydco	1,10-phen	2,2′-bipy	h					
1	0	0	1	4.47	—	4.47	96.2	3.7
1	0	0	2	7.38	—	2.91	40.3	2.0
1	0	0	3	8.13	—	0.75	22.1	2.0
0	1	0	1	4.54	—	4.54	87.5	2.0
0	1	0	2	8.76	—	4.22	17.9	2.0
0	0	1	1	2.01	—	2.01	44.0	2.0
0	0	1	2	3.49	—	1.48	13.1	2.0
1	1	0	1	13.52	[2,5-pydcoH(1,10-phen)]/[2,5-pydcoH][1,10-phen]	9.05	32.6	4.6
1	1	0	2	17.85	[2,5-pydcoH_2_(1,10-phen)]/[2,5-pydcoH_2_][1,10-phen]	10.47	52.3	4.0
1	1	0	3	21.36	[2,5-pydcoH_3_(1,10-phen)]/[2,5-pydcoH_3_][1,10-phen]	13.23	34.0	2.9–3.1
1	1	0	4	27.92	[2,5-pydcoH_3_(1,10-phenH)]/[2,5-pydcoH_3_][1,10-phenH]	15.25	25.7	2.5
2	1	0	3	32.14	[2,5-pydcoH_2_(2,5-pydcoH)(1,10-phen)]/[2,5-pydcoH_2_][2,5-pydcoH][1,10-phen]	20.29	16.6	4.3
1	2	0	4	34.37	[2,5-pydcoH_2_(1,10-phenH)_2_]/[2,5-pydcoH_2_][1,10-phenH]_2_	17.91	8.9	4.8
1	0	1	0	9.24	[2,5-pydco(2,2-bipy)]/[2,5-pydco][2,2-bipy]	—	9.5	6.2–12.0
1	0	1	2	11.31	[2,5-pydcoH_2_(2,2-bipy)]/[2,5-pydcoH_2_][2,2-bipy]	3.93	33.2	3.7
1	0	1	3	15.85	[2,5-pydcoH_3_(2,2-bipy)]/[2,5-pydcoH_3_][2,2-bipy]	7.72	46.2	2.3–2.6
2	0	1	2	24.16	[(2,5-pydcoH)_2_(2,2-bipy)]/[2,5-pydcoH]_2_[2,2-bipy]	15.22	24.9	4.8
2	0	1	4	26.74	[(2,5-pydcoH_2_)_2_(2,2-bipy)]/[2,5-pydcoH_2_]_2_[2,2-bipy]	11.98	16.6	3.6
2	0	1	6	29.12	[(2,5-pydcoH_3_)_2_(2,2-bipy)]/[2,5-pydcoH_3_]_2_[2,2-bipy]	12.86	9.3	2.2
1	0	2	3	32.63	[2,5-pydcoH_3_(2,2-bipy)_2_]/[2,5-pydcoH_3_][2,2-bipy]_2_	24.50	8.0	3.7
1	0	2	4	34.87	[2,5-pydcoH_2_(2,2-bipyH)_2_]/[2,5-pydcoH_2_][2,2-bipyH]_2_	23.47	3.6	2.1

**Fig. 19:**
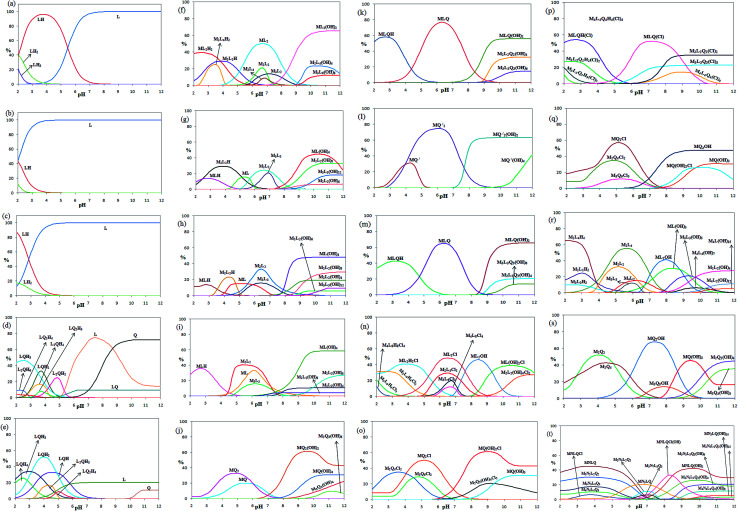
(*contd.*)

The pH titration data in the absence of metal ions were used to get the protonation constants for L and Q (K_*n*_^H^ [H_*m*_L]/[H_(*m*−*n*)_L][H]^*n*^, the charges are omitted for simplicity) *via* the program BEST.^49^ The corresponding distribution diagrams for 2,5-pydco (a), 2,2′-bipyridine (b), 1,10-phenanthroline (c), 2,5-pydco/2,2′-bipyridine (d), 2,5-pydco/1,10-phenanthroline (e), 2,5-pydco/Co^2+^ [1 : 2] (f), 2,5-pydco/Co^2+^ [1 : 1] (g), 2,5-pydco/Ni^2+^ (h), 2,5-pydco/Zn^2+^ (i), 2,2′-bipyridine/Co^2+^ (j), 2,5-pydco/2,2′-bipyridine/Co^2+^ (k), 1,10-phenanthroline/Co^2+^ (l), 2,5-pydco/1,10-phenanthroline/Co^2+^ (m), 2,5-pydco/Cu^2+^ (n), 2,2′-bipyridine/Cu^2+^ (o), 2,5-pydco/2,2′-bipyridine/Cu^2+^ (p), 1,10-phenanthroline/Cu^2+^ (q), 2,5-pydco/Ag^+^ (r), 1,10-phenanthroline/Ag^+^ (s), 2,5-pydco/1,10-phenanthroline/Ag^+^/Cu^2+^ (t) are shown in Fig. 19 (see also Table 5).

**Table:** Overall stability constants of 2,5-pydco/2,2-bipyridine or 1,10-phenanthroline/M^*n*+^/N^+^ (l/q/m/n) binary, ternary and quaternary systems in aqueous solution at 25 °C *μ* = 0.1 M of KNO_3_

System	m	n	l	q	h	cl	log *β*	Max%	At pH
Co^2+^–2,5-pydco [1 : 2]	1	0	2	0	0		17.23	49.9	6.5–6.9
	1	0	2	0	2		19.74	39.4	2.5
	1	0	2	0	−2		−2.16	65.3	11.1–12.0
	2	0	2	0	0		23.84	21.0	6.7
	2	0	2	0	1		25.28	29.0	3.9
	2	0	4	0	0		29.97	8.9	6.8
	2	0	4	0	2		31.10	24.9	4.3
	2	0	4	0	−4		7.02	23.4	10.3
	3	0	2	0	0		33.76	13.9	7.0–7.3
	3	0	6	0	−6		3.12	12.4	11.4–12.0
Co^2+^–2,5-pydco [1 : 1]	1	0	1	0	0		10.49	15.7	5.3
	1	0	1	0	1		13.21	13.9	2.8
	1	0	1	0	−4		−21.05	44.7	10.3
	2	0	2	0	0		23.79	24.4	6.6
	2	0	2	0	1		25.30	29	3.9
	2	0	2	0	−6		1.94	6.2	11.1–12.0
	2	0	2	0	−8		−9.62	33.1	10.8–11.2
	3	0	3	0	0		34.98	20.7	6.9
	3	0	3	0	−12		−28.96	18.3	11.5–12.0
Ni^2+^–2,5-pydco	1	0	1	0	0		11.01	15.1	5.0
	1	0	1	0	1		13.85	13.9	3.1
	1	0	1	0	−4		−19.96	48.1	9.9–12.0
	2	0	2	0	0		23.04	33.2	6.4
	2	0	2	0	1		25.00	23.3	4.3
	2	0	2	0	−4		3.11	19.4	10.4–12.0
	2	0	2	0	−6		−2.02	5.9	11.2–12.0
	2	0	2	0	−8		−10.17	29.8	117–12.0
	3	0	3	0	0		34.82	15.9	6.3–6.5
	3	0	3	0	−12		−27.84	9.5	10.8–12.0
Zn^2+^–2,5-pydco	1	0	1	0	0		11.92	33.2	6.1
	1	0	1	0	1		13.97	36.0	2.8
	1	0	1	0	−4		−18.46	58.4	10.2–12.0
	2	0	2	0	0		23.99	40.5	5.4
	2	0	2	0	−4		2.89	10.6	9.8–12.0
	2	0	2	0	−6		−3.14	4.2	8.0–12.0
	2	0	2	0	−8		−10.98	27.0	12.0
	3	0	3	0	0		34.25	15.8	6.2
Co^2+^–2,2-bipyridine	1	0	0	1	0		12.91	20.0	5.4
	1	0	0	1	−4		−16.59	30.9	10.8–11.2
	1	0	0	2	0		20.88	32.6	4.5–4.9
	1	0	0	2	−2		−0.63	61.4	9.6
	2	0	0	3	−6		2.39	9.5	11.2
	2	0	0	4	−4		8.56	22.4	12.0
Co^2+^–2,5-pydco–2,2-bipyridine	1	0	1	1	0		22.53	76.8	6.3
	1	0	1	1	1		25.18	57.9	2.7
	1	0	1	1	−2		6.01	55.7	10.8–12.0
	2	0	2	2	−4		8.47	32.5	11.4–12.0
	3	0	3	3	−6		10.35	14.5	11.7–12.0
Co^2+^–1,10-phenanthroline	1	0	0	1	0		13.89	31.1	4.1–4.3
	1	0	0	1	−4		−18.03	41.6	12.0
	1	0	0	2	0		25.46	74.7	6.0
	1	0	0	2	−2		−2.38	63.3	10.0–12.0
Co^2+^–2,5-pydco–1,10-phenanthroline	1	0	1	1	0		23.15	64.9	6.2
	1	0	1	1	1		25.07	42.5	3.2
	1	0	1	1	−2		4.52	65.4	10.9–12.0
	2	0	2	2	−4		6.31	20.6	11.4–12.0
	3	0	3	3	−6		7.95	13.9	11.8–12.0
Cu^2+^–2,5-pydco	1	0	1	0	−2	1	2.46	39.0	10.4–10.7
	1	0	2	0	0	1	28.83	48.4	6.5
	1	0	2	0	−1	0	13.41	46.4	8.4
	1	0	2	0	2	1	31.60	40.9	3.9
	2	0	2	0	−4	2	11.43	27.5	12.0
	2	0	4	0	0	2	34.27	29.5	6.4–6.6
	2	0	4	0	4	2	39.76	31.3	2.7
	3	0	6	0	0	3	46.01	19.7	6.7
	3	0	6	0	6	3	48.89	20.9	2.0
	4	0	8	0	0	4	52.93	12.0	6.7
	4	0	8	0	8	4	55.02	13.1	2.0
Cu^2+^–2,2-bipyridine	1	0	0	1	−3	0	−10.71	30.9	10.8–11.2
	1	0	0	1	−2	1	−4.63	61.4	9.0
	1	0	0	2	0	1	29.58	50.2	5.1
	2	0	0	2	−4	2	13.03	20.6	9.0–9.3
	2	0	0	4	0	2	36.82	35.1	3.6
	3	0	0	6	0	3	47.12	29.2	4.8
Cu^2+^–2,5-pydco–2,2-bipyridine	1	0	1	1	0	1	32.69	52.3	7.2
	1	0	1	1	1	1	33.88	54.2	2.7
	2	0	2	2	0	2	41.86	35.0	9.7–12.0
	2	0	2	2	2	2	43.94	27.4	2.4–2.6
	3	0	3	3	0	3	49.97	22.9	10.3–12.0
	3	0	3	3	3	3	52.35	19.9	2.0
	4	0	4	4	0	4	54.71	14.3	8.9
	4	0	4	4	4	4	57.99	11.4	2.0
Cu^2+^–1,10-phenanthroline	1	0	0	1	−3	0	−10.83	30.9	10.8–11.2
	1	0	0	1	−2	1	−3.29	26.3	10.1
	1	0	0	2	−1	0	12.74	47.6	9.7–12.0
	1	0	0	2	0	1	29.96	57.1	5.1
	2	0	0	4	0	2	37.21	34.9	4.8
	3	0	0	6	0	3	47.48	11.6	5.3
Ag^+^–2,5-pydco	0	1	1	0	−3		−11.26	30.4	8.3
	0	1	2	0	−1		12.50	41.1	7.9–8.1
	0	2	2	0	0		25.11	32.5	5.0–5.2
	0	2	2	0	2		27.19	24.6	3.0
	0	2	2	0	−4		−1.04	27.7	12.0
	0	2	4	0	0		32.81	55.2	5.6
	0	2	4	0	4		36.09	65.3	2.0
	0	3	3	0	0		39.98	12.0	6.0
	0	3	3	0	3		42.19	10.1	2.3
	0	3	3	0	−5		9.67	21.2	9.3
	0	4	1	0	−15		−18.34	5.1	12.0
	0	4	2	0	0		41.13	15.3	6.2
	0	4	2	0	−12		−12.69	11.0	12.0
	0	4	4	0	−7		8.36	6.2	9.8
Ag^+^–1,10-phenanthroline	0	1	0	1	−3		−14.08	45.6	9.4
	0	1	0	2	−1		13.77	67.8	7.3
	0	2	0	2	0		27.06	51.9	4.0
	0	2	0	2	−4		−0.79	45.0	12.0
	0	2	0	4	0		33.23	42.5	4.5
	0	3	0	4	−3		17.18	35.6	11.6–12.0
	0	3	0	5	−1		29.19	13.9	7.8
Ag^+^–Cu^2+^–2,5-pydco–1,10-phenanthroline	1	1	1	1	0		39.83	45.3	3.7
	1	1	1	1	0	1	44.98	6.6	3.7
	1	1	1	1	−2		18.06	42.3	9.6
	1	1	1	1	−1	1	20.17	33.2	8.3
	2	2	2	2	0		52.14	30.4	3.5–3.9
	2	2	2	2	−4		19.92	26.1	9.9
	3	3	3	3	0		59.79	17.4	3.9
	3	3	3	3	−6		21.06	20.6	11.1–12.0
	4	4	4	4	0		63.18	10.2	3.9
	4	4	4	4	−8		25.89	11.4	11.3–12.0
	1	3	1	1	0		48.72	20.3	6.7
	1	3	1	1	−10		14.8	5.4	11.5–12.0
	2	5	2	2	0		59.99	11.1	7.0
	2	5	2	2	−15		9.87	3.0	11.4
	3	7	3	3	0		65.24	6.2	6.9
	3	7	3	3	−19		5.82	Neg.	12.0

As it is obvious from Table 5, the main species in the Co^2+^ (M) with 2,5-pydco (L) binary system (Fig. 19f and g) are ML_2_(OH)_2_ (at pH 11.1–12.0; 65.3%), ML_2_ (at pH 6.5–6.9; 49.9%), ML(OH)_4_ (at pH 10.3; 44.7%), ML_2_H_2_ 39.4% at pH 2.5 and M_2_L_2_(OH)_8_ at pH 10.8–11.2 (33.1%). In the case of Ni^2+^ (M) with 2,5-pydco (L) in the binary system, the most abundant species are ML(OH)_4_ existed at pH 9.9–12.0 by an extent of 48.1% and M_2_L_2_ (33.2% at pH 6.4). For the Zn^2+^—2,5-pydco binary system (Fig. 19i), ML(OH)_4_ (58.4% at pH 10.2–12.0), M_2_L_2_ (40.5% at pH 5.4) and MLH (36.0% at pH 2.8) are the most abundant species. For the Co^2+^ (M) with 2,2′-bipy (Q) in the binary system (Fig. 19j), the main species are MQ_2_(OH)_2_ at pH 9.6 by an extent of 61.4% and MQ_2_ with an extent of 32.6% existed at pH 4.5–4.9. In the Co^2+^ (M) with 1,10-phen (Q′) for the binary system, the most abundant species are MQ_2_ existed at pH 6.0 by an extent of 74.7% and MQ_2_(OH)_2_ (63.3% at pH 10.0–12.0). In the case of Co^2+^ with L and Q in the ternary complexes system, the most abundant species are MLQ existed at pH 6.3 by an extent of 76.8%, MLQH (57.9% at pH 2.7) and MLQ(OH)_2_ at pH 10.8–12.0 with 55.7%. For the Co^2+^ with L and Q′ in the ternary complexes system, the major species are MLQ(OH)_2_ existed at pH 10.9–12.0 by an extent of 65.4%, MLQ at pH 6.2 by an extent of 64.9% and MLQH (42.5% at pH 3.2).

In the case of Cu^2+^ (M) with 2,5-pydco (L) in the binary system, the most abundant species are ML_2_Cl existed at pH 6.5 by an extent of 48.4%, ML_2_OH (46.4% at pH 8.4), ML_2_H_2_Cl (40.9% at pH 3.9) and ML(OH)_2_Cl (39.0% at pH 10.4–10.7). For the Cu^2+^ (M) with 2,2′-bipy (Q) in the binary system, the main species are MQ (OH)_2_Cl at pH 9.0 by an extent of 61.4%, MQ_2_Cl with an extent of 50.2% existed at pH 5.1 and M_2_Q_4_Cl_2_ (35.1% at pH 3.6). In the Cu^2+^ (M) with 1,10-phen (Q′) in the binary system, the major species are MQ_2_Cl (57.1% at pH 5.1), MQ_2_OH (47.6% at pH 9.7–12.0) and M_2_Q_4_Cl_2_ (34.9% at pH 4.8). In the case of Cu^2+^ with L and Q in the ternary complexes system, the most abundant species are MLQHCl at pH 2.7 by an extent of 54.2%, MLQCl (52.3% at pH 7.2) and M_2_L_2_Q_2_Cl_2_ at pH 9.7–12.0 with 35.0%. In the case of Ag^+^–2,5-pydco binary system, the main species are N_2_L_4_H_4_ at pH 2.0 by an extent of 65.3% N_2_L_4_ (55.2% at pH 5.6) and NL_2_OH with an extent of 41.1% existed at pH 7.9–8.1. In the Ag^+^ (M) with 1,10-phen (Q′) in the binary system, the major species are NQ_2_OH (67.8% at pH 7.3), N_2_Q_2_ (51.9% at pH 4.0), NQ(OH)_3_ (45.6% at pH 9.4), N_2_Q_2_(OH)_4_ (45.0% at pH 12.0) and N_2_Q_4_ (42.5% at pH 4.5). In the case of Ag^+^–Cu^2+^–2,5-pydco–1,10-phenanthroline quaternary system (M/N/L/Q), the most abundant species are MNLQ (45.3% at pH 3.7), MNLQ(OH)_2_ (42.3% at pH 9.6), MNLQ(OH)Cl (33.2% at pH 8.3), M_2_N_2_L_2_Q_2_ (30.4% at pH 3.5–3.9) and M_2_N_2_L_2_Q_2_(OH)_4_ (26.1% at pH 9.9). A comparison between the stoichiometry of the crystalline complexes and that of the most abundant species detected in solution revealed that they are very similar to those reported for the corresponding isolated complexes in the solid state.

## Conclusions

In summary, we have successfully synthesized and modified the H_2_pydco ligand from the N/O-donor ligand H_2_pydc in high yield by a new procedure to create a new O-only donor set than subsequent synthesis of the chlorinated derivative of this ligand. The results indicate that altering the donor set of the ligand profoundly affects the molecular and supramolecular structures of the corresponding transition metal complexes. The H_2_pydco ligand exhibits exceptional abilities to link metallic centers *via* its versatile chelating and bridging coordination modes. In this regard, we synthesized nine complexes with different architectures. In these complexes, H_2_pydco acts as a bidentate ligand through one oxygen atom of the carboxylate group and one oxygen atom of the *N*-oxide group. The formation of 5-membered chelate rings by the H_2_pydc ligand frequently resulted in complexes with planar conformations, while the H_2_pydco ligand formed complexes with 6-membered chelate rings displaying twisted conformations. *N*-oxide functionalization of the pyridine ring of the ligand led to the formation of complex and interesting supramolecular frameworks. The present study demonstrates that in these complexes cooperativity between various strong hydrogen bonds and a range of relatively weak π⋯π, C–H⋯π and C–O⋯π interactions is necessary for the construction of the supramolecular frameworks. DFT studies combined with MEP and NCI plot computational tools have been used to characterize and rationalize the O⋯π–hole interaction that is energetically very weak due to the small π–hole over the carboxylate group. Such π–hole appears due to the participation of the COO^−^ group in strong H-bonding interactions. The formation of binary, ternary and quaternary complexes in solution with stoichiometries very close to those of the solid state is strongly supported by the results of the potentiometric pH titration studies in aqueous solutions and the stoichiometry of the most abundant species in the solution was very similar to the corresponding crystalline complexes.

## Conflicts of interest

There are no conflicts to declare.

## Supplementary Material

RA-009-C9RA05143K-s001

RA-009-C9RA05143K-s002

## References

[cit1] Mirzaei M., Eshtiagh-Hosseini H., Bauza A., Zarghami S., Ballester P., Mague J. T., Frontera A. (2014). CrystEngComm.

[cit2] Mirzaei M., Eshtiagh-Hosseini H., Chahkandi M., Alfi N., Shokrollahi A., Shokrollahi N., Janiak A. (2012). CrystEngComm.

[cit3] Dogan D., Colak A. T., Sahin O., Tunc T., Celik O. (2015). Polyhedron.

[cit4] Lehn J. M. , Supramolecular chemistry: Concepts and perspectives, VCH, Weinheim, 1995

[cit5] Wang D., Tian Z. F., Wang F., Wen L. L., Li D. F. (2009). J. Inorg. Organomet. Polym..

[cit6] Mirzaei M., Aghabozorg H., Eshtiagh-Hosseini H. (2011). J. Iran. Chem. Soc..

[cit7] Fink G. S., Cuervo L. G., Therrien B., Evans H. S., Shulpin G. B. (2004). Inorg. Chim. Acta.

[cit8] Colak A. T., Colak F., Akduman D., Yesilel O. Z., Buyukgungor O. (2009). Solid State Sci..

[cit9] Kamatchi T. S., Chitrapriya N., Lee H., Fronczek C. F., Fronczekc F. R., Natarajan K. (2012). Dalton Trans..

[cit10] Kita E., Marai H., Zajac K. (2008). Transition Met. Chem..

[cit11] Sun L. P., Niu S. Y., Jin J., Yang G. D., Ye L. (2006). Eur. J. Inorg. Chem..

[cit12] Zhang D. J., Song T. Y., Wang L., Shi J., Xu J. N., Wang Y., Ma K. R., Yin W. R., Zhang L. R., Fan Y. (2009). Inorg. Chim. Acta.

[cit13] Mautner F. A., Albering J. H., Vicente R., Andrepont C., Gautreaux J. G., Gallo A. A., Massoud S. S. (2013). Polyhedron.

[cit14] Wibowo A. C., Vaughn S. A., Smith M. D., zur Loye H. C. (2010). Inorg. Chem..

[cit15] Mahata P., Natarajan S. (2005). Eur. J. Inorg. Chem..

[cit16] Mirzaei M., Eshtiagh-Hosseini H., Karrabi Z., Molcanov K., Eydizadeh E., Mague J. T., Bauza A., Frontera A. (2014). CrystEngComm.

[cit17] Mirzaei M., Eshtiagh-Hosseini H., Shamsipur M., Saeedi M., Ardalani M., Bauza A., Mague J. T., Frontera A., Habibi M. (2015). RSC Adv..

[cit18] Shahbazi M., Mehrzad F., Mirzaei M., Eshtiagh-Hosseini H., Mague J. T., Ardalani M., Shamsipur M. (2017). Inorg. Chim. Acta.

[cit19] Mirzaei M., Eshtiagh-Hosseini H., Bazargan M., Mehrzad F., Shahbazi M., Mague J. T., Bauza A., Frontera A. (2015). Inorg. Chim. Acta.

[cit20] Bazargan M., Mirzaei M., Eshtiagh-Hosseini H., Mague J. T., Bauza A., Frontera A. (2016). Inorg. Chim. Acta.

[cit21] Xue L., Luo F., Che Y. X., Zheng J. M. (2007). J. Mol. Struct..

[cit22] Shankar K., Das B., Baruah J. B. (2013). RSC Adv..

[cit23] Wei Y., Hou H., Li L., Fan Y., Zhu Y. (2005). Cryst. Growth Des..

[cit24] Huang S. L., Zhang L., Lin Y. J., Jin G. X. (2013). CrystEngComm.

[cit25] Wen L. L., Lu Z. D., Ren X. M., Duan C. Y., Meng Q. J., Gao S. (2009). Cryst. Growth Des..

[cit26] Wen L. L., Dang D. B., Duan C. Y., Li Y. Z., Tian Z. F., Meng Q. J. (2005). Inorg. Chem..

[cit27] Sun H. L., Wang X. L., Jia L., Cao W., Wang K. Z., Du M. (2012). CrystEngComm.

[cit28] Xiong Y., Fan Y. Z., Yang R., Chen S., Pan M., Jiang J. J., Su C. Y. (2014). Chem. Commun..

[cit29] Balzarini J., Stevens M., De Clercq E., Schols D., Pannecouque C. (2005). J. Antimicrob. Chemother..

[cit30] Lis S., Hnatejko Z., Barczynski P., Elbanowski M. (2002). J. Alloys Compd..

[cit31] X–AREA: Program for the Acquisition and Analysis of Data, Version 1.30, Stoe & Cie GmbH, Darmstadt, Germany, 2005

[cit32] X–RED: Program for Data Reduction and Absorption Correction, Version 1.28b, Stoe & Cie GmbH, Darmstadt, Germany, 2005

[cit33] X–SHAPE: Program for Crystal Optimization for Numerical Absorption Correction, Version 2.05, Stoe & Cie GmbH, Darmstadt, Germany, 2004

[cit34] Sheldrick G. M. , SHELXS97. Program for Crystal Structure Solution, University of Göttingen, Germany, 1997

[cit35] Sheldrick G. M. , SHELXL97. Program for Crystal Structure Refinement, University of Göttingen, Germany, 1997

[cit36] Prince E. and Wilson A. J. C., International Tables for X-ray Crystallography, vol. C, 1995

[cit37] X-STEP32: Crystallographic Package, Version 1.07b, Stoe & Cie GmbH, Darmstadt, Germany, 2000

[cit38] Çolak A. T., Çolak F., Yeşilel O. Z., Akduman D., Yılmaz F., Tümer M. (2010). Inorg. Chim. Acta.

[cit39] Sheshmani S., Aghabozorg H., Ghadermazi M. (2007). Acta Crystallogr., Sect. E: Struct. Rep. Online.

[cit40] Aghabozorg H., Derikvand Z., Nemati A., Ghadermazi M. (2007). Acta Crystallogr., Sect. E: Struct. Rep. Online.

[cit41] Aghabozorg H., Derikvand Z., Nemati A., Bahrami Z., Attar Gharamaleki J. (2008). Acta Crystallogr., Sect. E: Struct. Rep. Online.

[cit42] Frisch M. J. , Trucks G. W., Schlegel H. B., Scuseria G. E., Robb M. A., Cheeseman J. R., Scalmani G., Barone V., Petersson G. A., Nakatsuji H., Li X., Caricato M., Marenich A., Bloino J., Janesko B. G., Gomperts R., Mennucci B., Hratchian H. P., Ortiz J. V., Izmaylov A. F., Sonnenberg J. L., Williams-Young D., Ding F., Lipparini F., Egidi F., Goings J., Peng B., Petrone A., Henderson T., Ranasinghe D., Zakrzewski V. G., Gao J., Rega N., Zheng G., Liang W., Hada M., Ehara M., Toyota K., Fukuda R., Hasegawa J., Ishida M., Nakajima T., Honda Y., Kitao O., Nakai H., Vreven T., Throssell K., Montgomery, Jr J. A., Peralta J. E., Ogliaro F., Bearpark M., Heyd J. J., Brothers E., Kudin K. N., Staroverov V. N., Keith T., Kobayashi R., Normand J., Raghavachari K., Rendell A., Burant J. C., Iyengar S. S., Tomasi J., Cossi M., Millam J. M., Klene M., Adamo C., Cammi R., Ochterski J. W., Martin R. L., Morokuma K., Farkas O., Foresman J. B. and Fox D. J., Gaussian 09, Revision C.02, Gaussian, Inc., Wallingford CT, 2016

[cit43] Grimme S., Antony J., Ehrlich S., Krieg H. (2010). J. Chem. Phys..

[cit44] Zhao Y., Truhlar D. G. (2008). Theor. Chem. Acc..

[cit45] Sadhukhan D., Maiti M., Pilet G., Bauzá A., Frontera A., Mitra S. (2015). Eur. J. Inorg. Chem..

[cit46] Boys S. B., Bernardi F. (1970). Mol. Phys..

[cit47] Contreras-García J., Johnson E. R., Keinan S., Chaudret R., Piquemal J. P., Beratan D. N., Yang W. (2011). J. Chem. Theory Comput..

[cit48] Johnson E. R., Keinan S., Mori-Sanchez P., Contreras-Garcia J., Cohen A. J., Yang W. (2010). J. Am. Chem. Soc..

[cit49] Martell E. and Motekaitis R. J., Determination and Use of Stability Constants, VCH, New York, 2nd edn, 1992

[cit50] Devereuxa M., McCann M., Leon V., Geraghty M., McKee V., Wikaira J. (2000). Polyhedron.

[cit51] Sweeton F. H., Mesmer R. E., Baes Jr C. F. (1974). J. Solution Chem..

[cit52] Miller C. W., Benson L. V. (1983). Water Resour. Res..

[cit53] Shahbazi M., Mehrzad F., Mirzaei M., Eshtiagh-Hosseini H., Mague J. T., Ardalani M., Shamsipur M. (2017). Inorg. Chim. Acta.

[cit54] Hendrickson J. B., Wang J. (2004). Org. Lett..

[cit55] Xia Z. Q., Wei Q., Chen S. P., Feng X. M., Xie G., Qiao C. F., Zhang G. C., Gao S. L. (2013). J. Solid State Chem..

[cit56] Nakamoto K. , Infrared and Raman Spectra of Inorganic and Coordination Compounds Part B: Applications in Coordination, Organometallic, and Bioinorganic Chemistry, Wiley, New York, 6th edn, 2009

[cit57] Arefian M., Mirzaei M., Eshtiagh-Hosseini H. (2018). J. Mol. Struct..

[cit58] Nishio M. (2004). CrystEngComm.

[cit59] Janiak C. A. (2000). J. Chem. Soc., Dalton Trans..

[cit60] Addison A. W., Rao T. N. (1984). J. Chem. Soc., Dalton Trans..

[cit61] Clarke R., Latham K., Rix C., Hobday M., White J. (2005). CrystEngComm.

[cit62] Eshtiagh-Hosseini H., Mirzaei M., Zarghami S., Bauza A., Frontera A., Mague J. T., Habibi M., Shamsipur M. (2014). CrystEngComm.

[cit63] Mahjoobizadeh M., Mirzaei M., Bauza A., Lippolis V., Aragoni M. C., Shamsipur M., Ghanbari M., Frontera A. (2016). ChemistrySelect.

[cit64] Mirzaei M., Eshtiagh-Hosseini H., Karrabi Z., Notash B., Bauza A., Frontera A. (2015). J. Mol. Struct..

[cit65] Eftekhar M., Mirzaei M., Hassanpoor A., Khosravi I., Bauza A., Mague J. T., Frontera A. (2015). J. Coord. Chem..

[cit66] Cattalini L., Cassol A., Marangoni G., Rizzardi G., Rotondo E. (1969). Inorg. Chim. Acta.

[cit67] Zhang W. M., Lu Z. M., Li W., Chen M., Cao D. M. (2012). Z. Kristallogr.–New Cryst. Struct..

[cit68] Shi F. N., Han Y. F., Liu C. B. (2012). J. Chem. Crystallogr..

[cit69] Shiu K. B., Chen Z. W., Liao F. L., Wang S. L. (2003). Acta Crystallogr., Sect. E: Struct. Rep. Online.

[cit70] Cremer D., Pople J. A. (1975). J. Am. Chem. Soc..

[cit71] Sattelle B. M., Almond A. (2012). Phys. Chem. Chem. Phys..

[cit72] Burgi H. B. (1975). Angew. Chem., Int. Ed..

[cit73] Bartlett G. J., Choudhary A., Raines R. T., Woolfson D. N. (2010). Nat. Chem. Biol..

[cit74] Bauza A., Frontera A., Mooibroek T. J. (2016). Cryst. Growth Des..

[cit75] Grabowski S. J. (2015). Molecules.

[cit76] Dutta D., Nath H., Frontera A., Bhattacharyya M. K. (2019). Inorg. Chim. Acta.

