# Supplementary material for: Effects of N-oxidation on the molecular and crystal structures and properties of isocinchomeronic acid, its metal complexes and their supramolecular architectures: experimental, CSD survey, solution and theoretical approaches
Source: RSC Adv. 2019 Aug 14;9(44):25382–404. doi: 10.1039/c9ra05143k (PMC9070054; doi:10.1039/c9ra05143k)
Supplement: RA-009-C9RA05143K-s001 [file RA-009-C9RA05143K-s001.pdf]

Electronic Supplementary Material (ESI) for RSC Advances.
This journal is © The Royal Society of Chemistry 2019

Supporting Information for

Effects of *N*-oxidation on the molecular and crystal structures and properties of the isocinchomeronic acid, its metal complexes and their supramolecular architectures: Experimental, CSD survey, solution and theoretical approaches

Zahra Hosseini-Hashemi,[a] Masoud Mirzaei,*,[a] Ameneh Jafari,[a] Peyman Hosseinpour,[a] Mohammad Yousefi,[b] Antonio Frontera,[c] Mahmoud Lari Dashtbayaz,[d] Mojtaba Shamsipur[e] and Mehdi Ardalani[e]

[a]*Department of Chemistry, Faculty of Science, Ferdowsi University of Mashhad, Mashhad, IRAN*

[b]*Department of Chemistry, Yadegar-e-Imam Khomeini (RAH) Shahr-e-Rey Branch, Islamic Azad University, Tehran, IRAN*

[c]*Department of chemistry, Universitat de les Illes Balears, Crta de Valldemossa km 7.5, 07122 Palma de Mallorca (Baleares), SPAIN*

[d]*Assistant Professor of Accounting, Ferdowsi University of Mashhad, Mashhad, IRAN*

[e]*Department of Analytical Chemistry, Razi University, Kermanshah, IRAN*

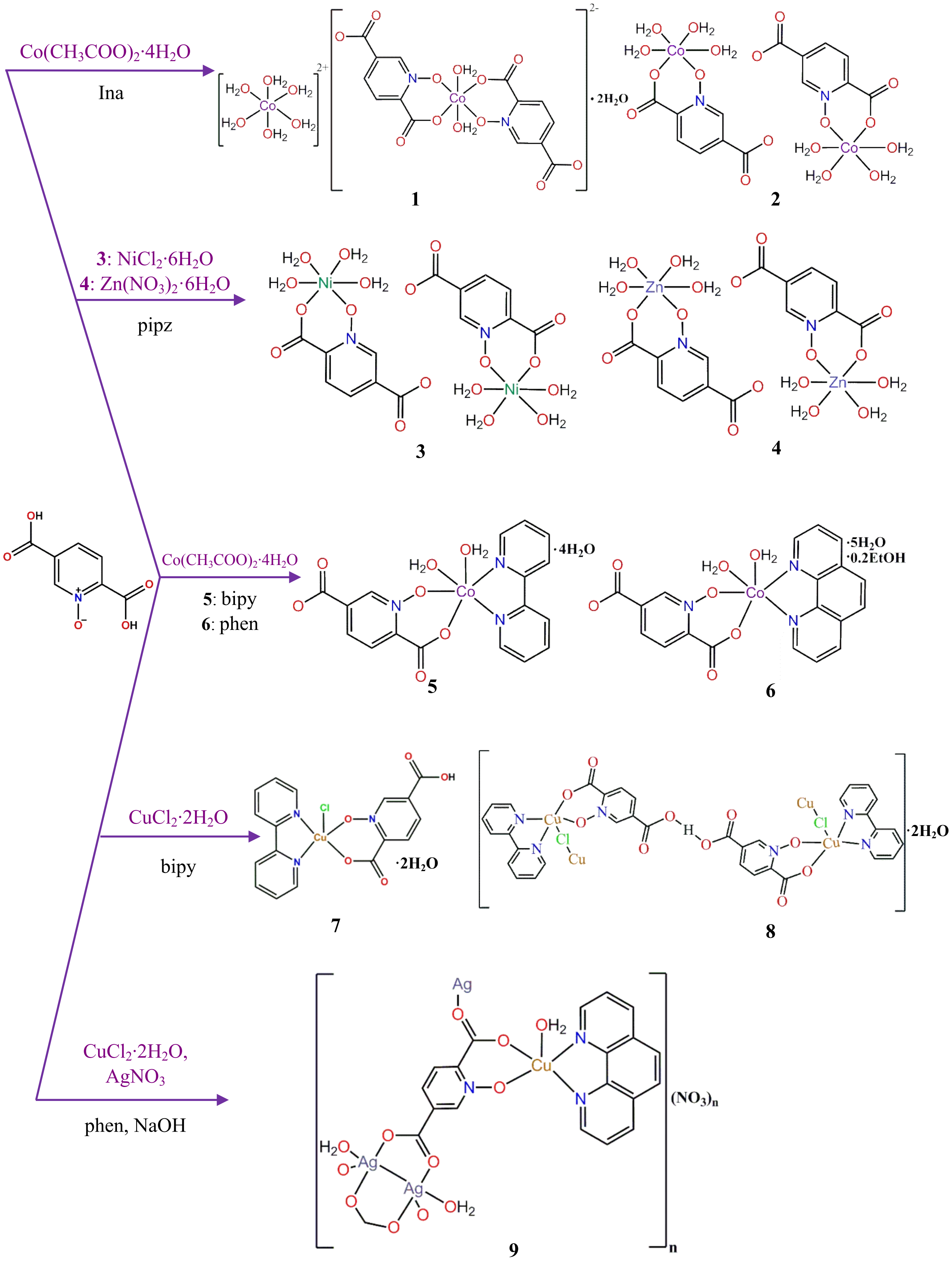

Scheme S1. Synthetic routes for compounds **1-9**.

[for reporting relative intensities, the intensity of the base peak in (see Fig. S1 in the Supporting Information) (94%) was changed to 100% and the intensities of the other peaks were corrected by a factor of 100/94].

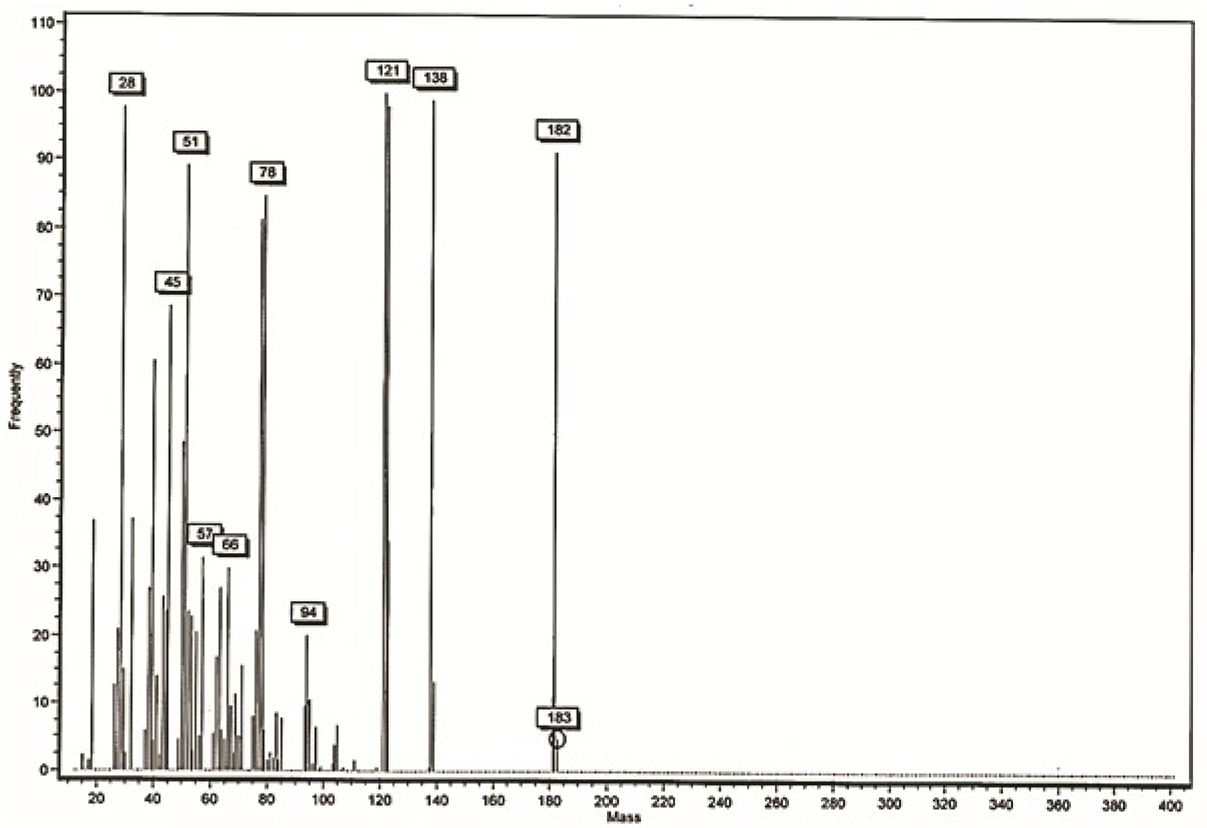

Fig. S1. Mass spectrum of H_2pydco.

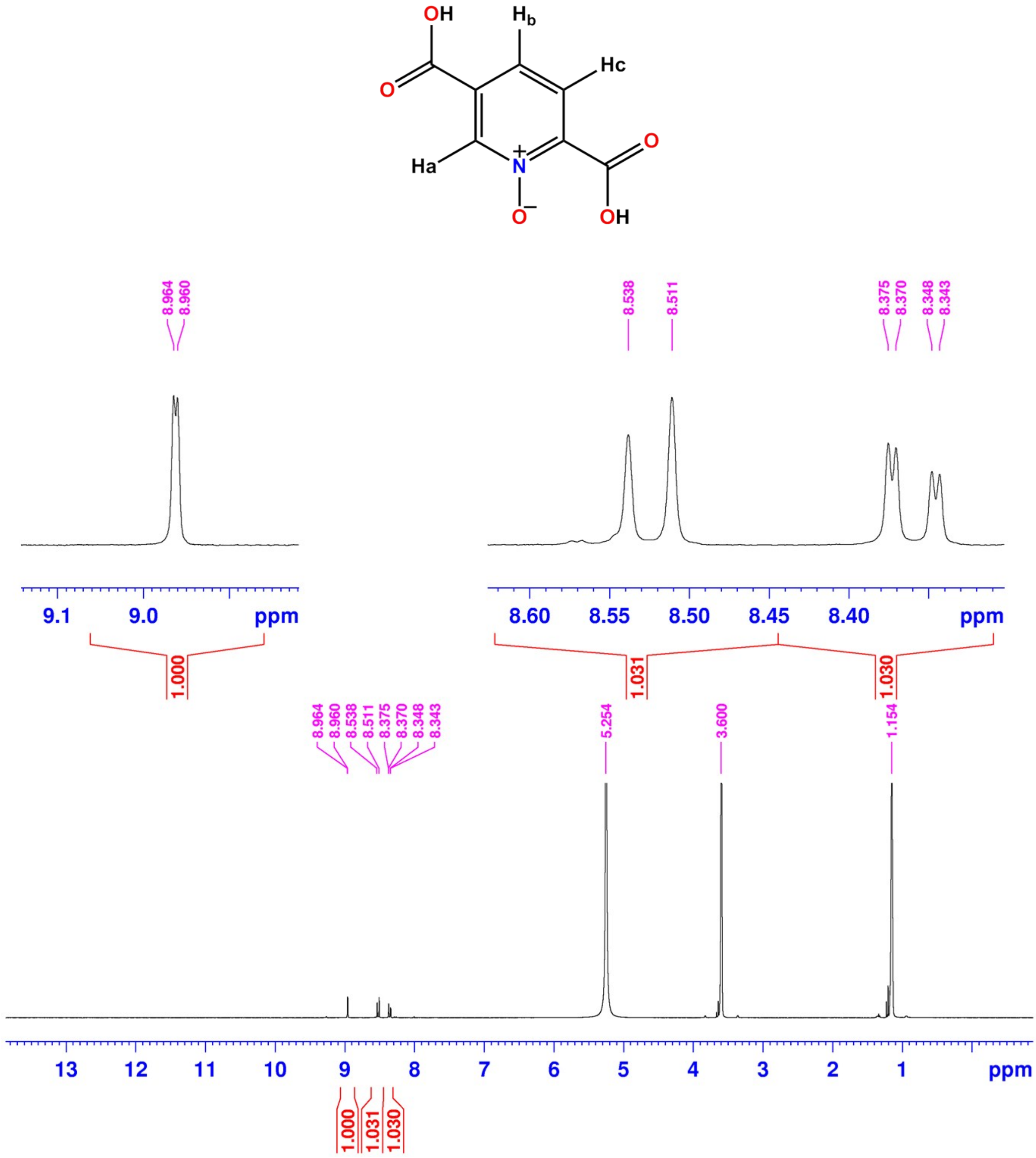

Fig. S2. ^{1}H NMR of H_2pydco.

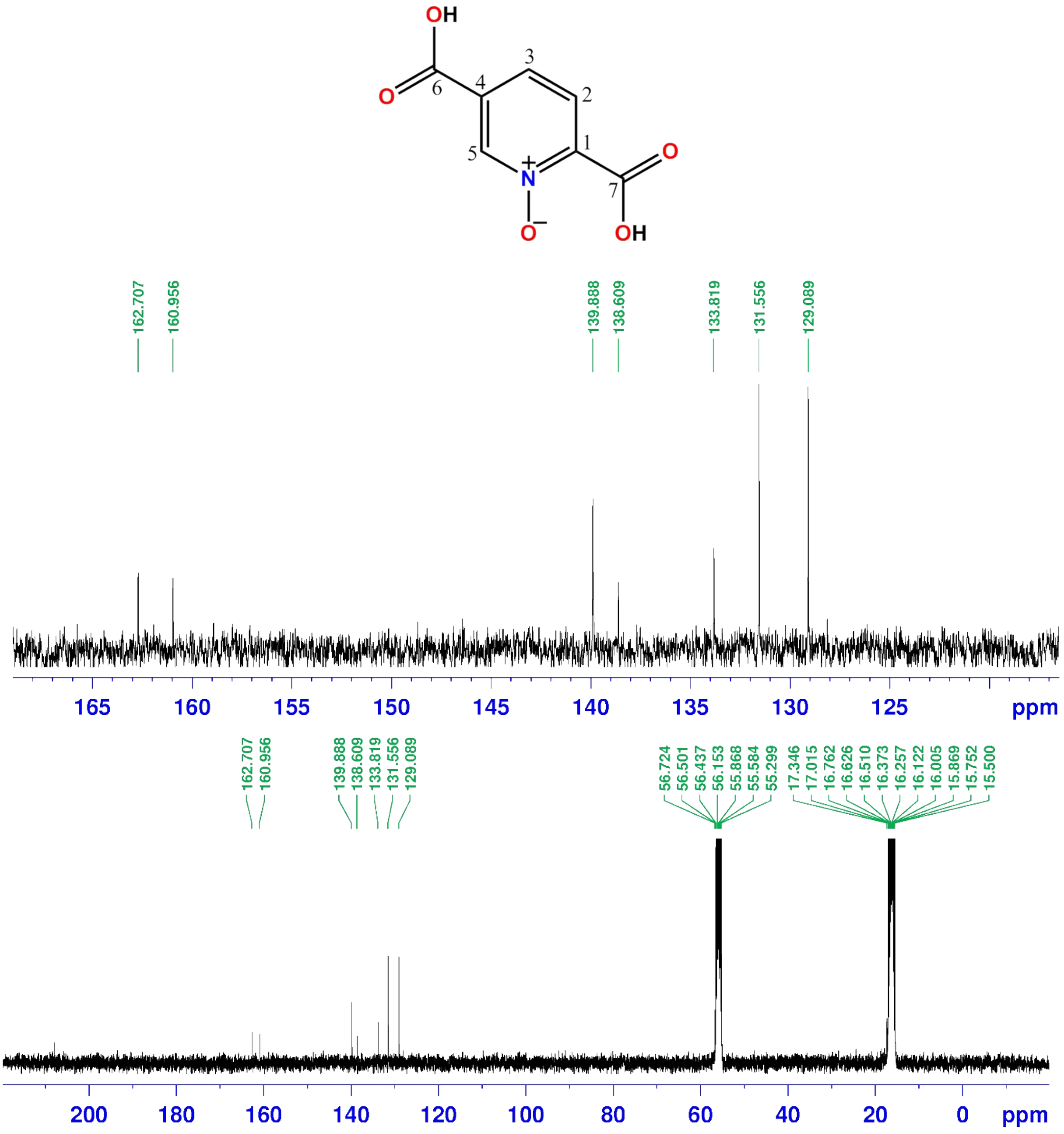

Fig. S3. $^{13}C\{^{1}H\}$ NMR spectrum of H_2pydco.

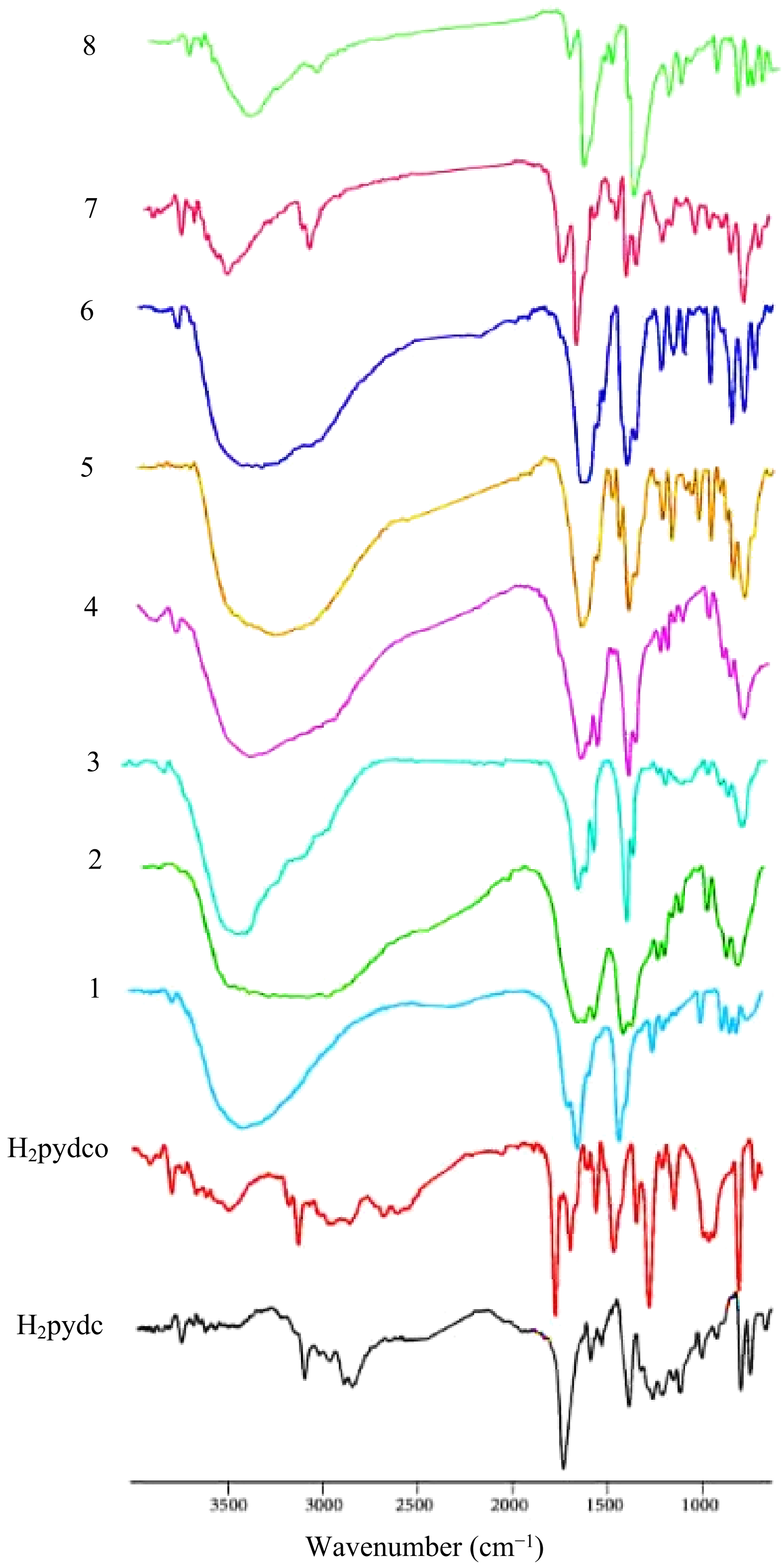

Fig. S4. Infrared spectra of H_2pydc, H_2pydco, and **1-9**.

Table S1. Selected bond lengths (Å) and valence angles (°) for **1–9**.

**1**			
Co1 O3i	2.018 (3)	O1 Co1 O6	89.4 (2)
Co1 O3	2.018 (3)	O3i Co1 O6i	92.77 (16)
Co1 O1i	2.032 (4)	O3 Co1 O6i	87.23 (16)
Co1 O1	2.032 (4)	O1i Co1 O6i	89.4 (2)
Co1 O6	2.116 (6)	O1 Co1 O6i	90.6 (2)
Co1 O6i	2.116 (6)	O6 Co1 O6i	180
Co2 O9	2.081 (4)	O9 Co2 O9ii	96.2 (3)
Co2 O9ii	2.081 (4)	O9 Co2 O8ii	87.2 (2)
Co2 O8ii	2.086 (6)	O9ii Co2 O8ii	87.4 (2)
Co2 O8	2.086 (6)	O9 Co2 O8	87.4 (2)
Co2 O7ii	2.097 (4)	O9ii Co2 O8	87.2 (2)
Co2 O7	2.097 (4)	O8ii Co2 O8	171.9 (2)
O3i Co1 O3	180	O9 Co2 O7ii	175.47 (18)
O3i Co1 O1i	86.17 (14)	O9ii Co2 O7ii	88.32 (18)
O3 Co1 O1i	93.83 (14)	O8ii Co2 O7ii	93.3 (2)
O3i Co1 O1	93.83 (14)	O8 Co2 O7ii	92.50 (19)
O3 Co1 O1	86.17 (14)	O9 Co2 O7	88.32 (18)
O1i Co1 O1	180	O9ii Co2 O7	175.47 (18)
O3i Co1 O6	87.23 (16)	O8ii Co2 O7	92.5 (2)
O3 Co1 O6	92.77 (16)	O8 Co2 O7	93.3 (2)
O1i Co1 O6	90.6 (2)	O7ii Co2 O7	87.2 (2)
Symmetry codes: (i) −x+1/2, −y+1/2, −z; (ii) −x+1, y, −z+1/2.			
**2**			
Co1 O10	2.0578(18)	Co2 O17	2.0714(18)
Co1 O8	2.0674(19)	Co2 O18	2.0738(17)
Co1 O5	2.0901(17)	Co2 O15	2.0756(17)
Co1 O7	2.0978(17)	Co2 O2	2.0873(17)
Co1 O1	2.0991(17)	Co2 O16	2.1039(19)
Co1 O9	2.1248(18)	Co2 O13	2.1041(17)
O10 Co1 O8	88.94(8)	O17 Co2 O18	179.41(8)
O10 Co1 O5	89.76(7)	O17 Co2 O15	93.74(7)
O8 Co1 O5	93.45(8)	O18 Co2 O15	85.69(7)
O10 Co1 O7	176.68(7)	O17 Co2 O2	85.74(7)
O8 Co1 O7	87.78(8)	O18 Co2 O2	94.10(8)
O5 Co1 O7	90.01(7)	O15 Co2 O2	91.77(7)
O10 Co1 O1	94.50(8)	O17 Co2 O16	86.27(8)
O8 Co1 O1	175.41(7)	O18 Co2 O16	93.93(8)

O5 Co1 O1	83.53(7)	O15 Co2 O16	92.55(8)
O7 Co1 O1	88.76(7)	O2 Co2 O16	171.15(6)
O10 Co1 O9	87.04(7)	O17 Co2 O13	90.30(7)
O8 Co1 O9	92.22(8)	O18 Co2 O13	90.25(7)
O5 Co1 O9	173.43(7)	O15 Co2 O13	174.82(7)
O7 Co1 O9	93.52(7)	O2 Co2 O13	85.31(7)
O1 Co1 O9	91.00(7)	O16 Co2 O13	90.94(8)
**3**			
Ni1 O6	2.0156(15)	Ni2 O16	2.0410(14)
Ni1 O7	2.0472(15)	Ni2 O18	2.0424(15)
Ni1 O9	2.0503(15)	Ni2 O10	2.0429(15)
Ni1 O4	2.0507(14)	Ni2 O15	2.0462(15)
Ni1 O1	2.0679(14)	Ni2 O13	2.0632(15)
Ni1 O8	2.1030(14)	Ni2 O17	2.0676(15)
O6 Ni1 O7	88.30(7)	O16 Ni2 O18	86.40(6)
O6 Ni1 O9	176.37(5)	O16 Ni2 O10	90.81(6)
O7 Ni1 O9	88.10(7)	O18 Ni2 O10	94.53(6)
O6 Ni1 O4	90.46(6)	O16 Ni2 O15	92.67(6)
O7 Ni1 O4	93.38(7)	O18 Ni2 O15	179.01(6)
O9 Ni1 O4	89.31(6)	O10 Ni2 O15	85.82(6)
O6 Ni1 O1	95.04(6)	O16 Ni2 O13	176.69(5)
O7 Ni1 O1	176.32(5)	O18 Ni2 O13	91.76(6)
O9 Ni1 O1	88.55(6)	O10 Ni2 O13	86.59(6)
O4 Ni1 O1	85.04(6)	O15 Ni2 O13	89.18(6)
O6 Ni1 O8	86.81(6)	O16 Ni2 O17	91.16(6)
O7 Ni1 O8	90.59(7)	O18 Ni2 O17	92.47(7)
O9 Ni1 O8	93.67(6)	O10 Ni2 O17	172.84(5)
O4 Ni1 O8	175.11(5)	O15 Ni2 O17	87.21(7)
O1 Ni1 O8	91.16(6)	O13 Ni2 O17	91.68(6)
**4**			
Zn1 O8	2.057(3)	Zn2 O18	2.037(3)
Zn1 O7	2.065(3)	Zn2 O16	2.062(3)
Zn1 O10	2.067(3)	Zn2 O15	2.072(3)
Zn1 O9	2.084(3)	Zn2 O13	2.111(3)
Zn1 O1	2.105(3)	Zn2 O17	2.129(3)
Zn1 O5	2.141(3)	Zn2 O2	2.145(3)
O8 Zn1 O7	85.85(12)	O18 Zn2 O16	89.39(13)
O8 Zn1 O10	95.02(12)	O18 Zn2 O15	177.41(11)
O7 Zn1 O10	178.75(13)	O16 Zn2 O15	88.24(12)
O8 Zn1 O9	93.45(12)	O18 Zn2 O13	89.04(12)

O7 Zn1 O9	94.31(12)	O16 Zn2 O13	94.22(12)
O10 Zn1 O9	86.55(12)	O15 Zn2 O13	90.10(11)
O8 Zn1 O1	92.25(11)	O18 Zn2 O17	86.72(12)
O7 Zn1 O1	94.18(12)	O16 Zn2 O17	92.47(13)
O10 Zn1 O1	84.89(11)	O15 Zn2 O17	94.42(11)
O9 Zn1 O1	170.10(11)	O13 Zn2 O17	172.04(11)
O8 Zn1 O5	173.72(12)	O18 Zn2 O2	94.42(12)
O7 Zn1 O5	89.40(12)	O16 Zn2 O2	174.93(11)
O10 Zn1 O5	89.67(12)	O15 Zn2 O2	87.89(11)
O9 Zn1 O5	90.99(11)	O13 Zn O2	82.52(10)
O1 Zn1 O5	84.00(10)	O17 Zn2 O2	91.08(11)
**5**			
Co1 O6	2.0782(16)	O1 Co1 O7	89.57(7)
Co1 O4	2.0829(17)	O6 Co1 N3	173.36(7)
Co1 O1	2.1009(17)	O4 Co1 N3	92.25(7)
Co1 O7	2.1019(17)	O1 Co1 N3	94.72(7)
Co1 N3	2.1072(17)	O7 Co1 N3	90.55(7)
Co1 N2	2.1153(18)	O6 Co1 N2	96.20(7)
O6 Co1 O4	90.02(7)	O4 Co1 N2	95.41(7)
O6 Co1 O1	91.72(7)	O1 Co1 N2	172.06(6)
O4 Co1 O1	84.01(7)	O7 Co1 N2	91.27(7)
O6 Co1 O7	87.87(7)	N3 Co1 N2	77.38(7)
O4 Co1 O7	173.18(6)		
**6**			
Co1 O1	2.095(2)	O1 Co1 O4	83.95(7)
Co1 O4	2.059(2)	O6 Co1 O1	92.47(6)
Co1 O6	2.113(2)	O4 Co1 N2	91.33(7)
Co1 O7	2.078(2)	O4 Co1 O7	173.25(7)
Co1 N2	2.133(2)	O1 Co1 N3	172.87(7)
Co1 N3	2.118(2)	O6 Co1 N2	172.93(7)
**7**			
Cu1 O4	1.922(3)	O4 Cu1 O1	90.15(13)
Cu1 O1	1.957(3)	O1 Cu1 N2	89.56(14)
Cu1 N2	1.955(3)	O4 Cu1 N3	91.14(14)
Cu1 N3	2.012(4)	N2 Cu1 N3	81.14(15)
Cu1 Cl1	2.4517(15)	O4 Cu1 Cl1	96.66(12)
O4 Cu1 N2	163.81(16)	O1 Cu1 Cl1	99.30(11)
O1 Cu1 N3	149.97(15)	N2 Cu1 Cl1	99.36(12)
N3 Cu1 Cl1	110.33(11)		

8			
Cu1−O4	1.927(2)	O4 Cu1 O1	89.07(8)
Cu1−O1	1.9414(17)	O4 Cu1 N3	91.47(9)
Cu1−N3	1.986(2)	O1 Cu1 N2	94.76(8)
Cu1−N2	1.999(2)	N3 Cu1 N2	81.22(9)
Cu1−Cl1	2.5553(7)	O4 Cu1 Cl1	97.94(7)
O1 Cu1 N3	166.23(7)	O1 Cu1 Cl1	95.26(6)
O4 Cu1 N2	164.24(8)	N2 Cu1 Cl1	96.92(6)
N3 Cu1 Cl1	98.29(6)		
**9**			
Ag1−O4	2.289(5)	Cu1−O1	1.932(3)
Ag1−O7	2.370(6)	Cu1−O2	1.907(4)
Ag1−Ag1	2.886(1)	Cu1−O6	2.236(4)
Ag1−O3	2.606(4)	Cu1−N2	2.003(4)
Ag1−O5	2.233(4)	Cu1−N3	1.996(4)
O4 Ag1 O7	85.8(2)	O1 Cu1 O2	92.9(1)
O4 Ag1 Ag1	77.0(1)	O1 Cu1 O6	95.0(2)
O4 Ag1 O3	91.8(1)	O1 Cu1 N2	159.7(2)
O4 Ag1 O5	146.7(1)	O1 Cu1 N3	89.5(2)
O7 Ag1 Ag1	162.7(2)	O2 Cu1 O6	97.4(2)
O7 Ag1 O3	90.5(2)	O2 Cu1 N2	90.2(2)
O7 Ag1 O5	115.7(2)	O2 Cu1 N3	166.0(2)
O3 Ag1 O5	112.0(1)	O6 Cu1 N2	104.5(2)
Ag1 Ag1 O3	87.94(9)	O6 Cu1 N3	96.1(2)
Ag1 Ag1 O5	80.79(9)	N2 Cu1 N3	82.9(2)

Table S2. Selected Hydrogen bond geometry, lengths (Å) and valence angles (°) for **1–9**.

D−H···A	D−H	H···A	D···A	D−H···A
**1**				
O10 H1···O4iii	0.89 (7)	1.90 (7)	2.769 (6)	165 (6)
O10 H2···O2iv	0.95 (9)	1.79 (9)	2.729 (5)	173 (8)
O6 H61···O10v	0.86 (7)	1.91 (7)	2.733 (6)	158 (7)
O6 H62···O5vi	0.78 (7)	2.07 (7)	2.837 (8)	166 (8)
O7 H71···O4i	0.81 (7)	2.00 (7)	2.759 (6)	155 (6)
O7 H72···O5vii	0.70 (7)	2.10 (7)	2.788 (5)	166 (7)
O8 H81···O1ii	0.91 (2)	2.35 (8)	3.012 (5)	130 (9)
O8 H81···O3viii	0.91 (2)	2.29 (6)	3.103 (6)	149 (10)
O8 H82···O10	0.91 (2)	1.92 (5)	2.755 (6)	152 (9)
O9 H91···O1ii	0.90 (7)	2.62 (7)	3.338 (7)	137 (5)
O9 H91···O2ii	0.90 (7)	1.92 (7)	2.796 (6)	165 (6)
O9 H92···O5ix	0.78 (7)	2.02 (7)	2.786 (6)	169 (8)

Symmetry codes: (i) −*x*+1/2, −*y*+1/2, −*z*; (ii) −*x*+1, *y*, −*z*+1/2; (iii) −*x*, −*y*, −*z*; (iv) −*x*+1/2, *y*+1/2, −*z*+1/2; (v) *x*, −*y*, *z*−1/2; (vi) *x*+1/2, *y*+1/2, *z*; (vii) *x*+1, −*y*, *z*+1/2; (viii) *x*+1/2, −*y*+1/2, *z*+1/2; (ix) −*x*+1/2, −*y*−1/2, −*z*.

**2**				
O7−H7A···O1	0.77(2)	2.12(3)	2.817(2)	150(2)
O7−H7B···O13	0.84(3)	1.89(3)	2.717(3)	172(3)
O8−H8A···O4	0.73(3)	2.13(3)	2.855(3)	178(4)
O8−H8B···O14	0.81(3)	2.01(3)	2.812(3)	169(3)
O9−H9A···O11	0.75(3)	1.94(3)	2.682(3)	173(3)
O9−H9B···O11	0.78(2)	2.12(2)	2.871(3)	162(3)
O10−H10A···O12	0.87(3)	1.79(3)	2.651(3)	171(3)
O10−H10B···O12	0.72(3)	2.10(3)	2.812(3)	168(3)
O15−H15A···O9	0.82	2.23	3.053(3)	177
O15−H15B···O4	0.88(3)	1.88(2)	2.765(3)	176(3)
O16−H16A···O6	0.78(3)	2.20(3)	2.957(3)	165(3)
O16−H16B···O1	0.74(3)	2.20(3)	2.933(3)	174(3)
O17−H17A···O5	0.78(3)	2.00(3)	2.762(3)	166(3)
O17−H17A···O6	0.78(3)	2.60(3)	3.221(3)	138(3)
O17−H17B···O2	0.79(3)	2.01(3)	2.754(3)	157(3)
O17−H17B···N2	0.79(3)	2.49(3)	3.005(3)	124(3)
O18−H18A···O3	0.77(3)	1.95(3)	2.685(3)	161(3)
O18−H18B···O3	0.86(3)	1.83(3)	2.686(3)	170(3)
C1−H1···O18	0.93	2.49	3.379(3)	159
C4−H4···O15	0.93	2.49	3.367(3)	157
C5−H5···O6	0.93	2.45	3.345(3)	162
C11−H11···O7	0.93	2.50	3.260(3)	139
C12−H12···O14	0.93	2.56	3.310(3)	139
**3**				
O6−H6A···O11	0.83	1.81	2.640(2)	175
O6−H6B···O11	0.79	2.02	2.798(2)	169
O7−H7A···O3	0.80	2.05	2.848(2)	178
O7−H7B···O14	0.82	2.01	2.821(2)	169
O8−H8A···O12	0.79	1.90	2.676(2)	170
O8−H8B···O12	0.77	2.14	2.890(2)	164
O9−H9A···O1	0.74	2.16	2.817(2)	150
O9−H9B···O13	0.83	1.90	2.720(2)	171
O15−H15A···O4	0.78	1.99	2.766(2)	174
O15−H15B···O10	0.82	2.01	2.770(2)	154
O15−H15B···N2	0.82	2.48	2.987(2)	121
O16−H16A···O8	0.83	2.24	3.031(2)	162
O16−H16B···O3	0.81	1.97	2.771(2)	176
O17−H17A···O5	0.76	2.19	2.941(2)	166
O17−H17B···O1	0.80	2.15	2.942(2)	172
O18−H18A···O2	0.88	1.81	2.683(2)	174
O18−H18B···O2	0.78	1.93	2.692(2)	165
C1−H1···O18	0.93	2.47	3.355(2)	159
C4−H4···O16	0.93	2.47	3.343(3)	157
C5−H5···O5	0.93	2.45	3.344(3)	162
C11−H11···O9	0.93	2.50	3.272(3)	141
C12−H12···O14	0.93	2.52	3.276(3)	139
**4**				
O8−H8A···O17	0.80(4)	2.29(4)	3.048(5)	159(4)
O8−H8B···O12	0.82(5)	1.93(5)	2.755(4)	175(5)
O9−H9A···O14	0.83(4)	2.09(4)	2.908(5)	172(3)
O9−H9B···O2	0.81(4)	2.12(4)	2.920(4)	167(4)
O10−H10A···O13	0.93(5)	1.82(5)	2.753(4)	174(5)
O10−H10A···O14	0.93(5)	2.57(5)	3.230(5)	129(4)
O10−H10B···O1	0.86(6)	2.54(5)	2.817(4)	100(4)
O10−H10B···O1	0.86(6)	1.95(5)	2.741(4)	152(5)

O10−H10B···N1	0.86(6)	2.46(5)	2.986(5)	120(4)
O15−H15A···O2	0.79(5)	2.05(5)	2.791(4)	158(6)
O15−H15A···N2	0.79(5)	2.54(5)	3.163(4)	137(5)
O15−H15B···O5	0.81(4)	1.91(4)	2.696(4)	165(4)
O16−H16A···O6	0.81(5)	2.00(5)	2.791(5)	164(5)
O16−H16B···O12	0.95(5)	1.90(5)	2.834(4)	167(4)
O17−H17A···O3	0.87(5)	1.82(5)	2.678(5)	167(5)
O17−H17B···O3	0.80(4)	2.07(4)	2.857(5)	167(5)
O18−H18A···O4	0.77(5)	1.88(5)	2.649(5)	176(6)
O18−H18B···O4	0.82(4)	1.99(5)	2.791(5)	168(4)
C4−H4···O15	0.93	2.51	3.271(5)	139
C5−H5···O6	0.93	2.57	3.329(6)	139
C8−H8···O7	0.93	2.47	3.359(5)	159
C11−H11···O8	0.93	2.48	3.362(5)	158
C12−H12···O14	0.93	2.47	3.366(6)	161
**5**				
O6−H6A···O2	0.84(4)	1.81(4)	2.653(2)	176(3)
O6−H6A···O11	0.81(4)	1.93(4)	2.736(3)	173(3)
O7−H7A···O3	0.77(4)	1.94(4)	2.717(3)	175(3)
O7−H7B···O9	0.83(2)	1.93(2)	2.752(3)	171(2)
O8−H8A···O2	0.91(3)	1.99(3)	2.877(4)	163(3)
O8−H8B···O5	0.93(2)	2.04(2)	2.932(4)	161(3)
O9−H9A···O3	0.83(3)	1.94(3)	2.768(3)	173(3)
O9−H9B···O11	0.84(3)	2.08(3)	2.916(4)	169(3)
O10−H10A···O1	0.85(2)	2.18(2)	2.970(3)	155(3)
O10−H10B···O5	0.87(2)	1.95(2)	2.814(3)	168(3)
O11−H11A···O8	0.86(2)	2.13(2)	2.944(4)	159(3)
O11−H11B···O10	0.86(3)	2.00(3)	2.821(4)	160(3)
C1−H1···O2	0.93	2.41	2.731(3)	100
C11−H11···O4	0.93	2.44	3.168(3)	135
**6**				
O7−H7A···O3	0.84(5)	1.86(5)	2.700(2)	175(4)
O7−H7B···O5	0.84(5)	1.88(5)	2.708(3)	169(4)
O8−H8A···O13	0.87	2.40	2.765(7)	105
O8−H8B···O11	0.87	1.90	2.740(6)	161
O10−H10A···O2	0.97(4)	1.80(4)	2.767(3)	173(5)
O10−H10B···O10	0.95(3)	2.40(4)	2.749(4)	101(2)
O11−H11A···O3	0.91(3)	1.87(3)	2.776(3)	175(3)
O11−H11B···O10	0.90(3)	1.93(3)	2.810(4)	169(3)
O12−H12A···O1	0.95(3)	1.80(3)	2.751(3)	178(3)
O12−H12A···N1	0.95(3)	2.46(3)	3.294(3)	147(3)
O12−H12B···O11	0.93(3)	1.88(3)	2.796(3)	168(3)
O13−H13A···O12	0.96(6)	1.84(7)	2.734(5)	154(3)
O13−H13B···O5	0.94(6)	2.43(7)	2.966(5)	116(5)
C5−H5···O13A	0.95	2.59	3.332(6)	135
C10−H10···O7	0.95	2.59	3.394(3)	142
C17−H17···O4	0.95	2.51	3.250(3)	135
C18−H18···O12	0.95	2.53	3.252(3)	133
**7**				
O2−H2···O6	0.86(6)	1.67(6)	2.526(5)	175(7)
O6−H6A···O3	0.87(3)	2.05(4)	2.838(7)	149(5)
O6−H6B···O7	0.88(5)	1.84(4)	2.709(7)	168(5)
O7−H7A···Cl1	0.76(6)	2.63(7)	3.176(5)	131(5)
O7−H7B···O5	0.88(6)	1.92(6)	2.794(6)	169(6)
C1−H1···O7	0.9300	2.48	3.345(7)	156
C10−H10···O5	0.9300	2.45	3.265(6)	146
C14−H14···O3	0.9300	2.53	3.371(6)	151
C15−H15···Cl1	0.9300	2.79	3.560(6)	141
**8**				

O2−H2···O2	1.2300	1.2300	2.463(3)	180
O6−H6A···O5	0.90(4)	1.98(4)	2.864(4)	170(4)
O6−H6B···O6	0.91(3)	2.59(3)	3.440(5)	156(4)
C1−H1···O6	0.9300	2.55	3.373(5)	147
C5−H5···O6	0.9300	2.30	3.109(5)	145
C11−H11···O3	0.9300	2.38	3.295(4)	170
C14−H14···O3	0.9300	2.39	3.282(4)	160
**9**				
O6−H6A···O8	0.76(7)	2.57(7)	3.256(13)	151(7)
O6−H6A···O9	0.76(7)	2.54(8)	3.26(2)	160(7)
O6−H6B···O5	0.78(7)	2.03(7)	2.803(6)	172(5)
O7−H7A···O8	0.88(6)	2.30(6)	3.108(13)	153(5)
O7−H7B···O10	0.86(6)	2.28(8)	2.96(3)	136(6)
C4−H4···O9	0.9300	2.54	3.176(15)	126
C13−H13···O8	0.9300	2.36	3.255(14)	162
C19−H19···O10	0.9300	2.39	3.09(3)	132

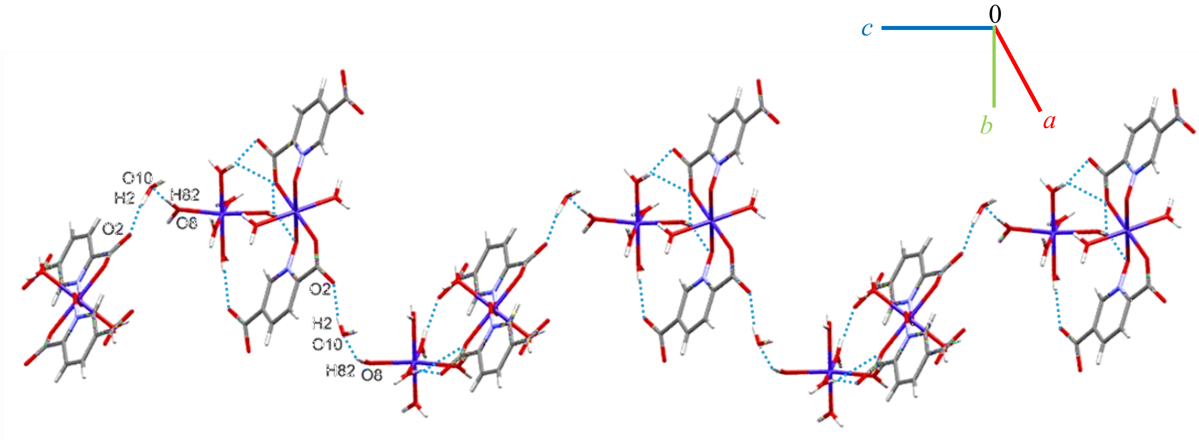

Fig. S5. Representation of the zigzag chain along the *c* axis in **1**.

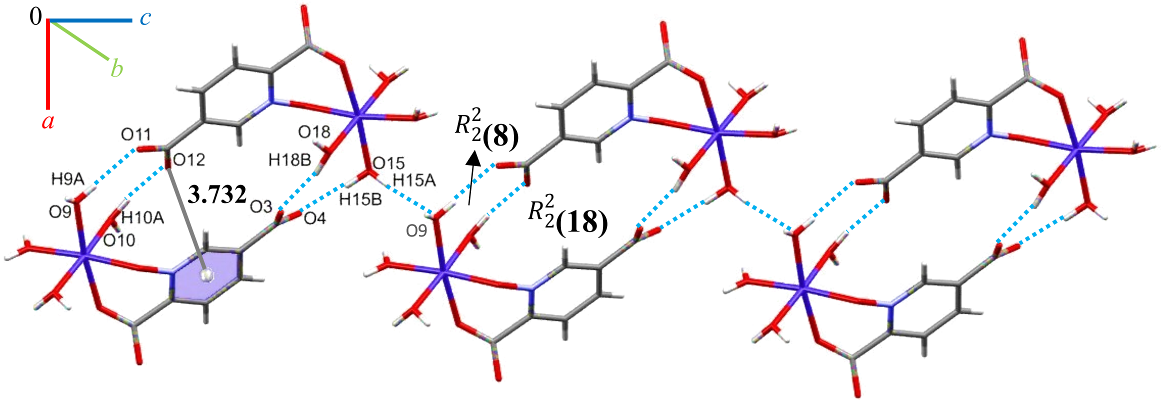

Fig. S6. Extended one-dimensional structure determined along the *c* by hydrogen bonding, C−O···π interactions and cyclic motifs in the crystal lattice of **2**.

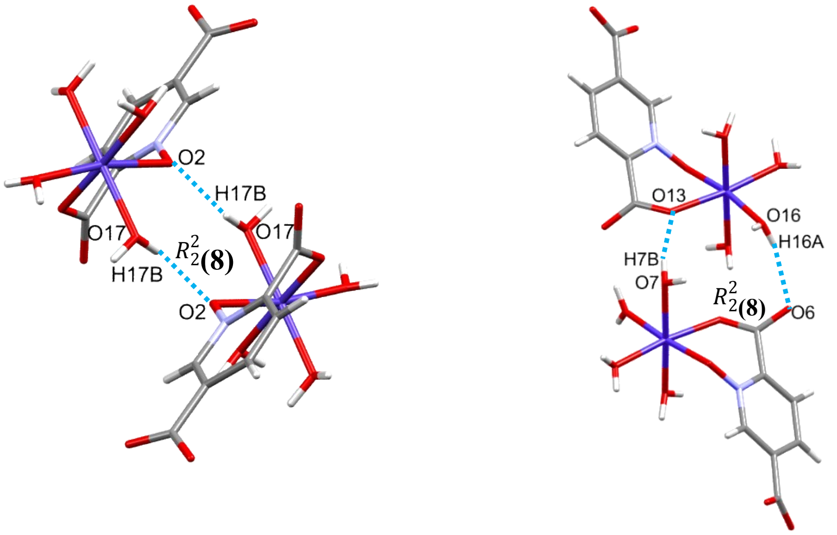

Fig. S7. Representation of two model cyclic motifs of $R_2^2(8)$ created by O−H···O hydrogen bonding along the *b* direction in compound **2**.

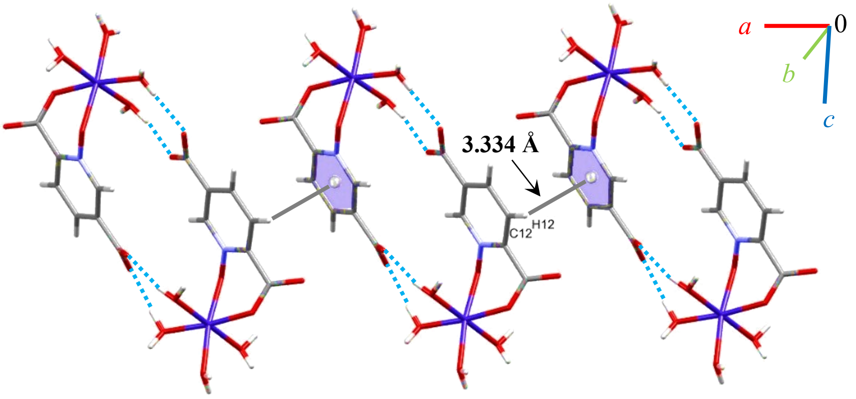

Fig. S8. Representation of the one-dimensional linear chain along the *a* axis by C−H···π interactions in **2**.

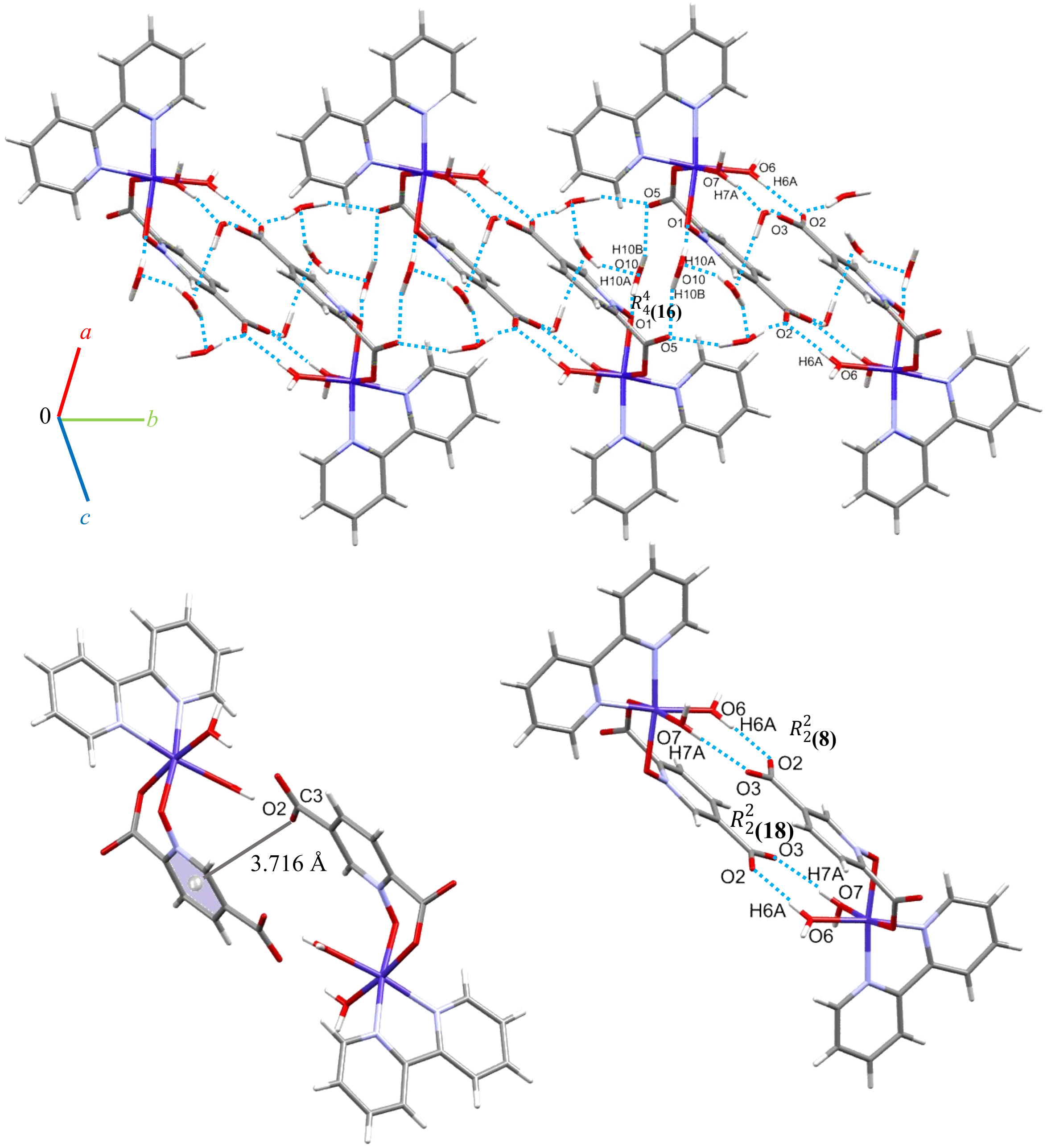

Fig. S9. Representation of the linear chain along the *b* axis by hydrogen bonding, C−H···*π* interactions in **5**.

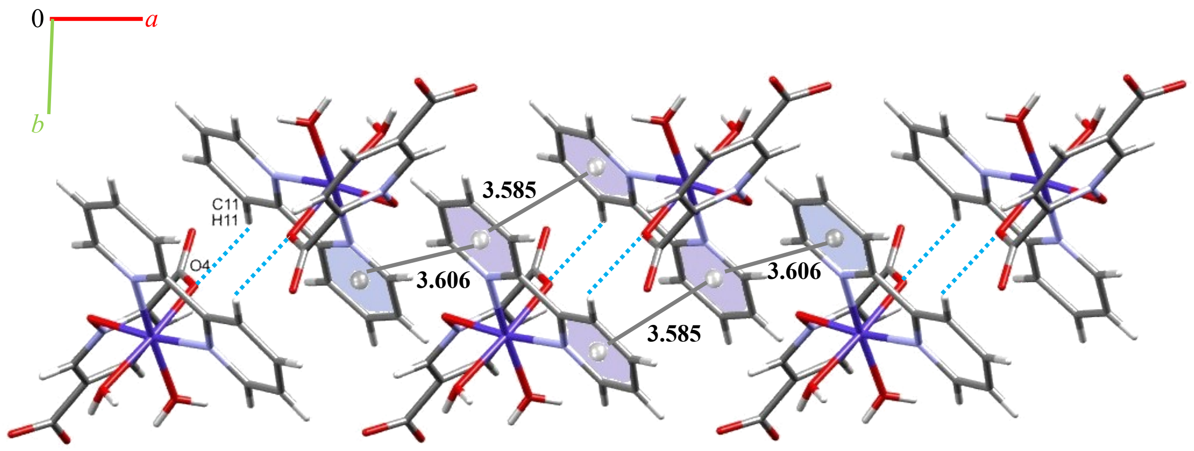

Fig. S10. Representation of the chain along the *a* axis formed by $\pi\cdots\pi$ interactions in **5**, Distances are shown in Å.

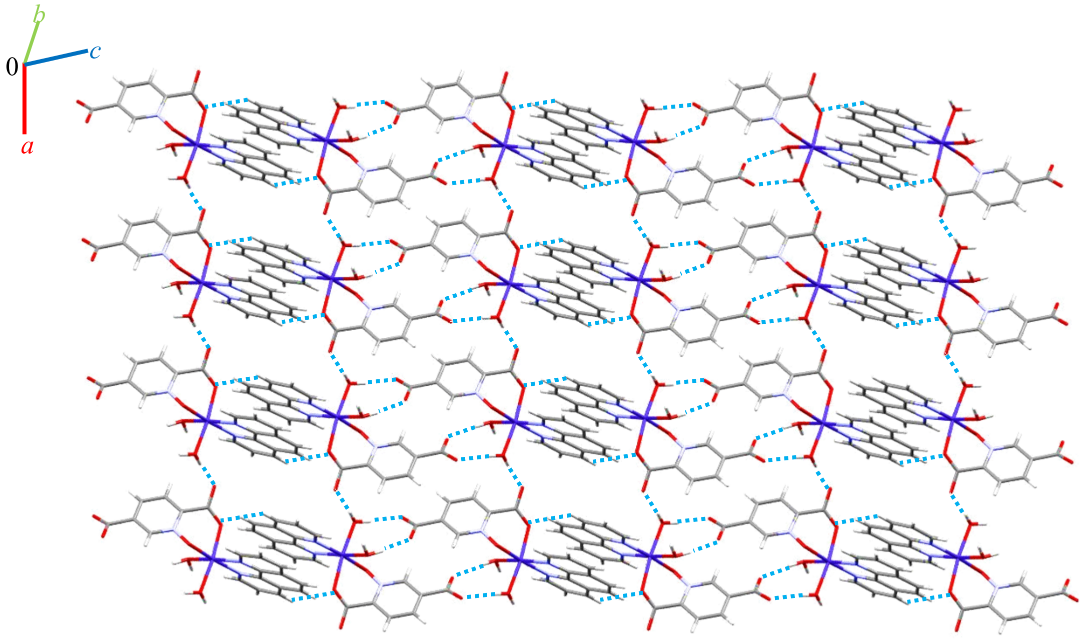

Fig. S11. Extended two-dimensional structure in **6**.

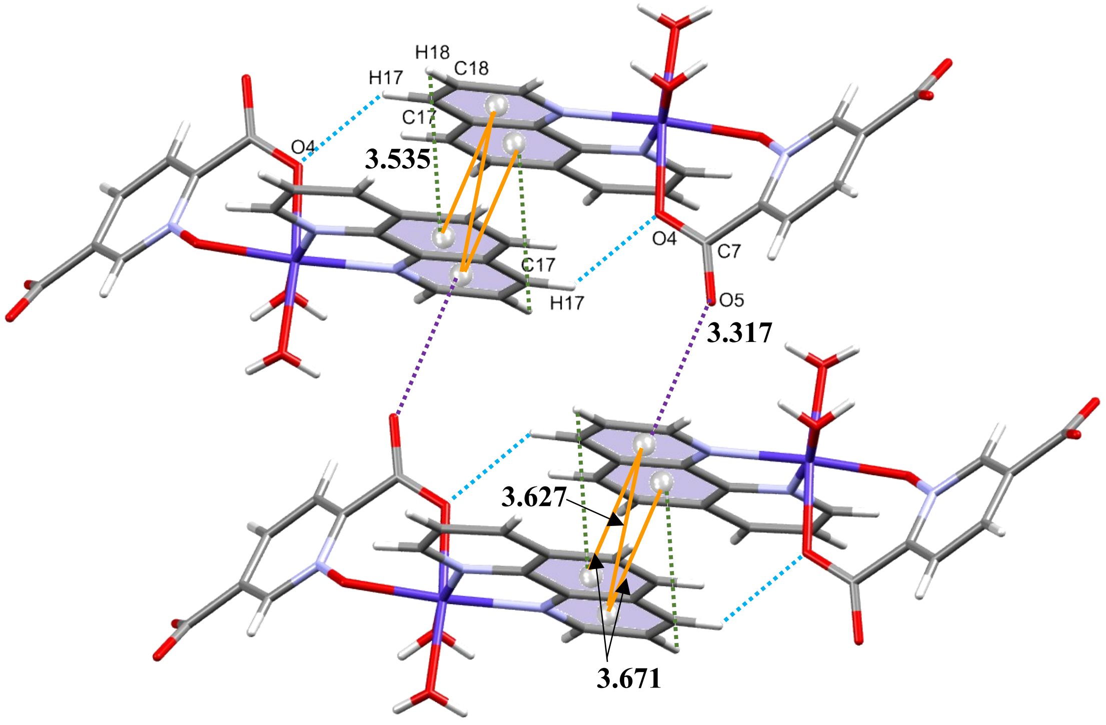

Fig. 12. Representation of $\pi\cdots\pi$, C−H⋯π, and C−O⋯π interactions in a two-dimensional sheet of **6**, Distances are shown in Å. The hydrogen bonds, C−H⋯π, and C−O⋯π interactions are shown as blue, orange, green, and purple dotted lines, respectively and $\pi\cdots\pi$ interactions are shown as orange dashed lines.

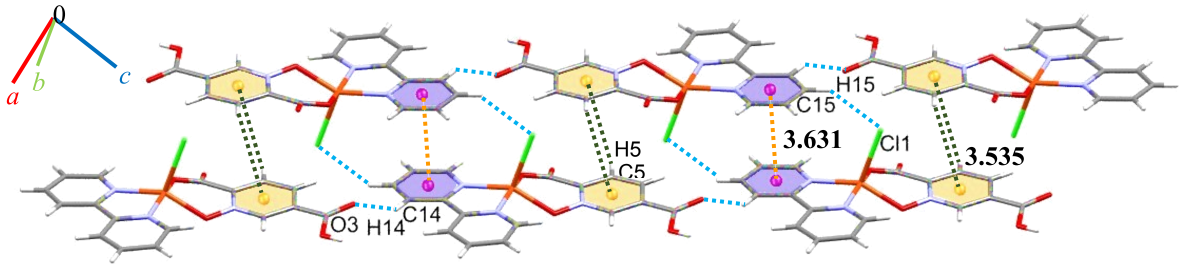

Fig. S13. Representation of the chain along side view in **7**, Distances are shown in Å. The hydrogen bonds, $\pi\cdots\pi$, and C−H⋯π interactions are shown as blue, orange, and green dotted lines, respectively.

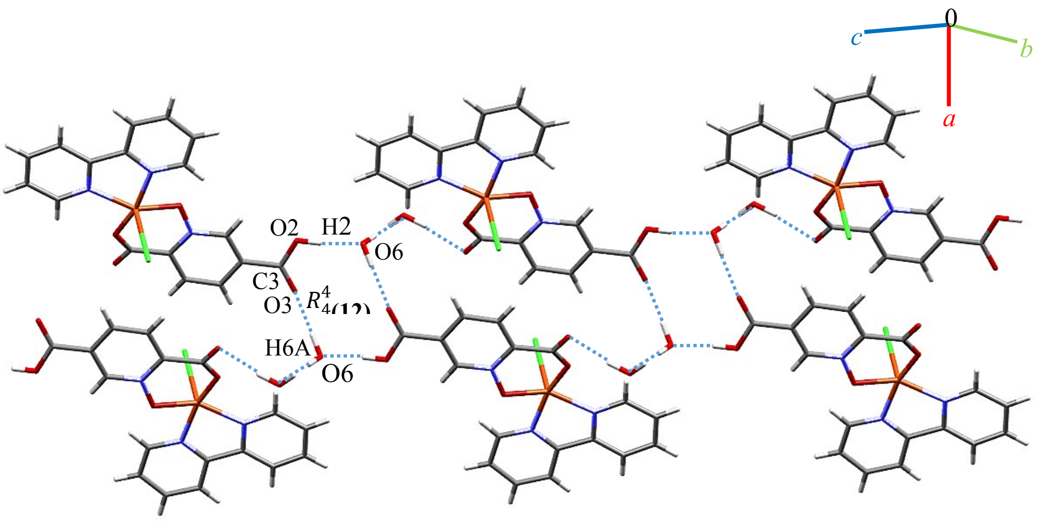

Fig. S14. Representation of the chain along side view formed *via* O−H···O hydrogen bonds in **7**.

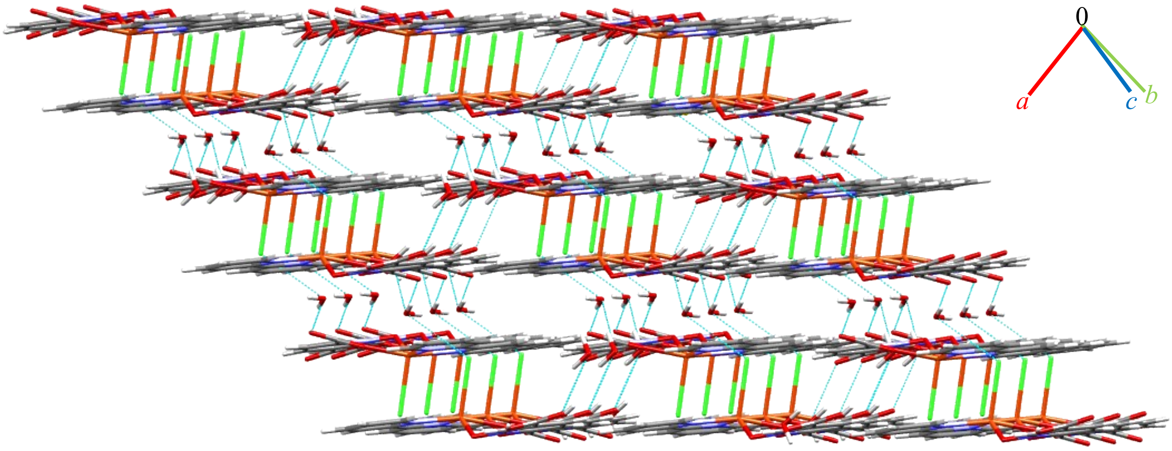

Fig. S15. Extended three-dimensional network of compound **7.**

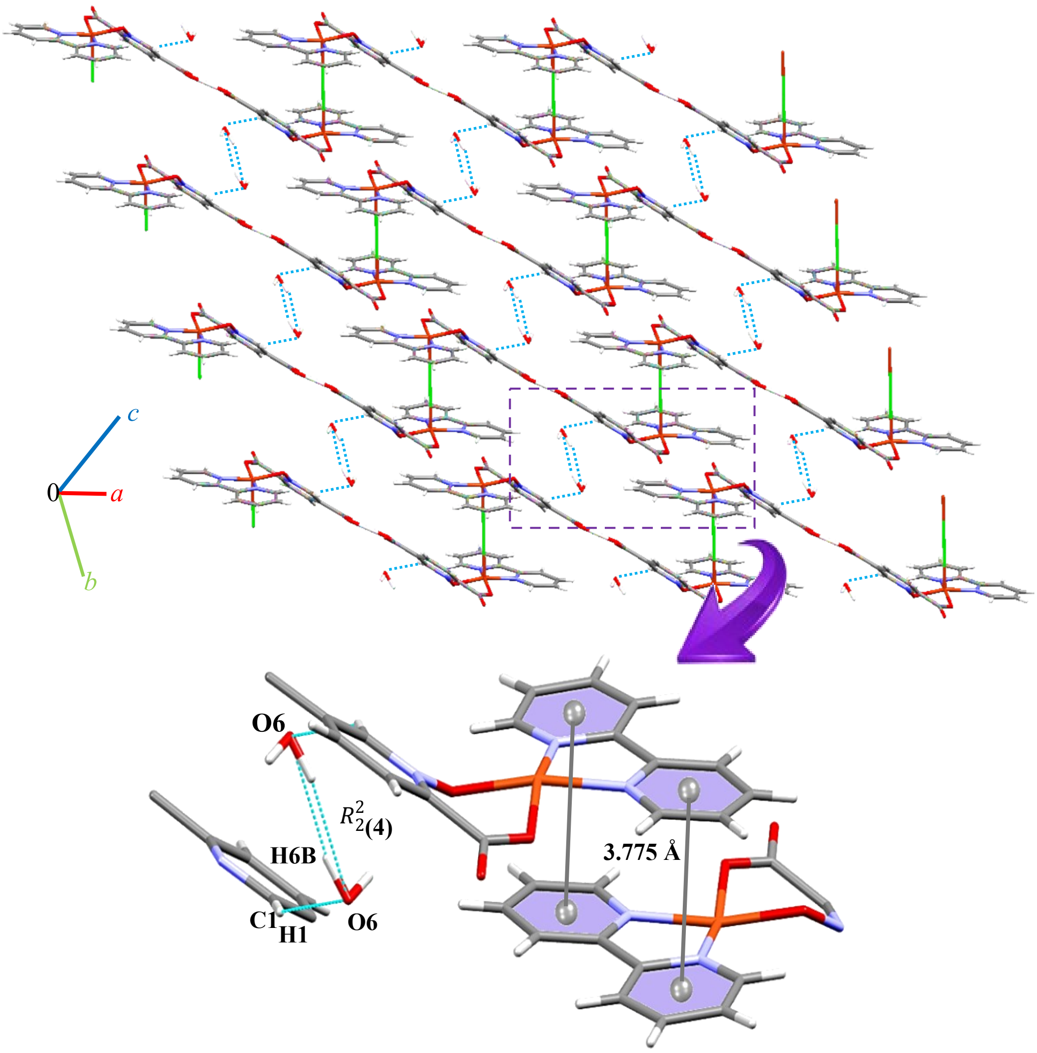

Fig. S16. Extended two-dimensional structure of **8** with cyclic $R_2^2(4)$ motifs formed *via* O−H···O hydrogen bonding and $\pi\cdots\pi$ interactions. The hydrogen bonds and $\pi\cdots\pi$ interactions are shown as blue dotted lines and gray dashed lines, respectively.

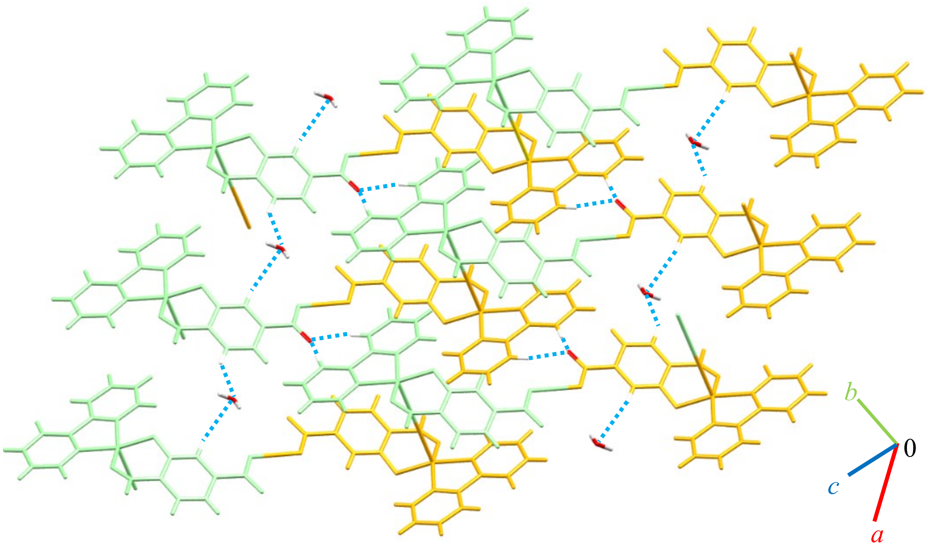

Fig. S17. Perspective view of the one dimensional chain of **8** along the *a* axis.

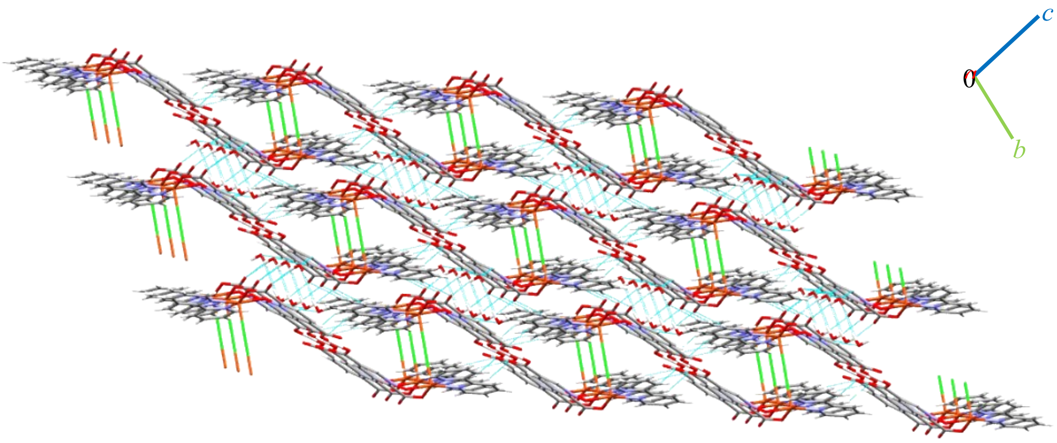

Fig. S18. Hydrogen bonding and $\pi\cdots\pi$ stacking forming the three-dimensional network of **8**.

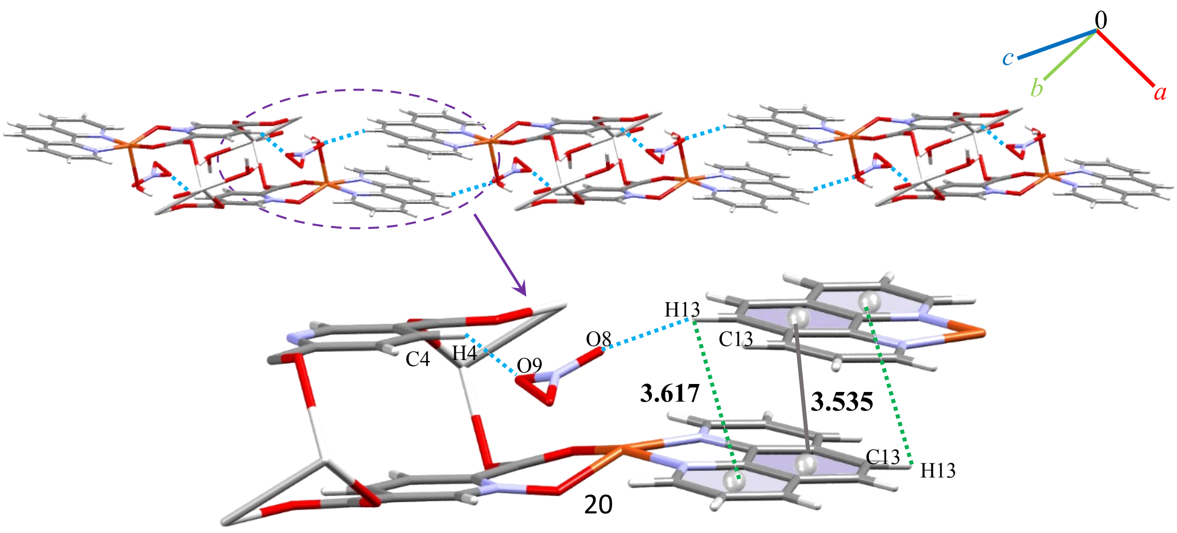

Fig. S19. Representation of the one-dimensional chain along side view by hydrogen bonding and $\pi\cdots\pi$ interactions in **9**, Distances are shown in Å. The hydrogen bonds, C−H$\cdots\pi$ and $\pi\cdots\pi$ interactions are shown as blue and green dotted lines and gray dashed lines, respectively.

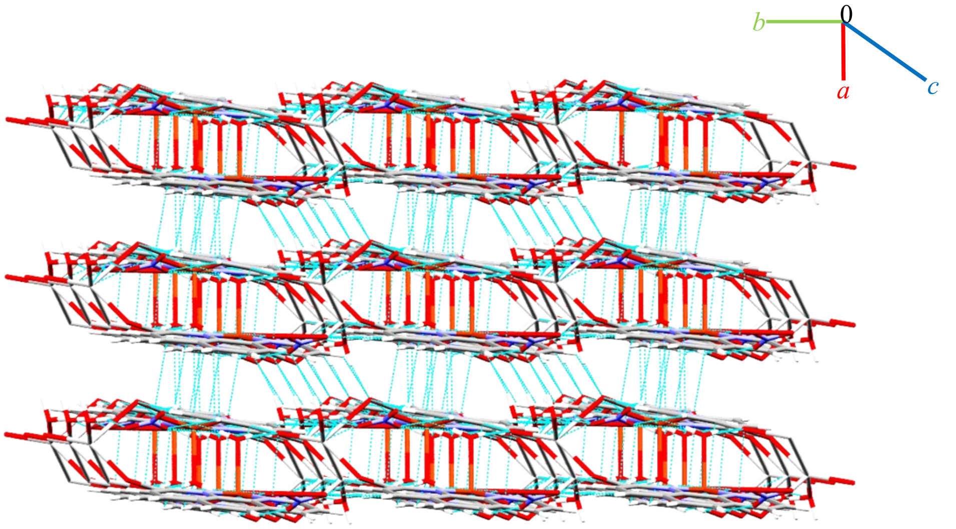

Fig. S20. One-dimensional chains link together by hydrogen bonds and weak interactions to form a three-dimensional network of **9**.

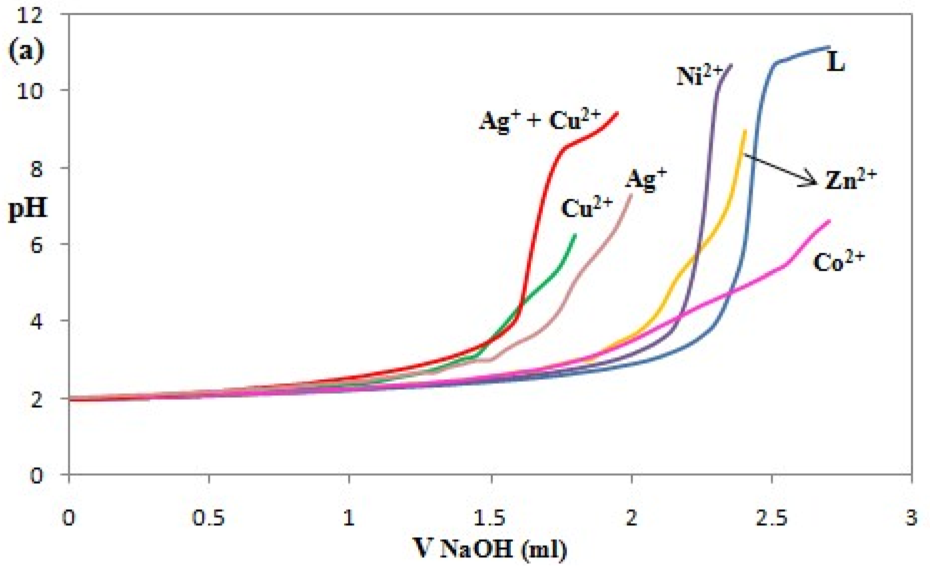

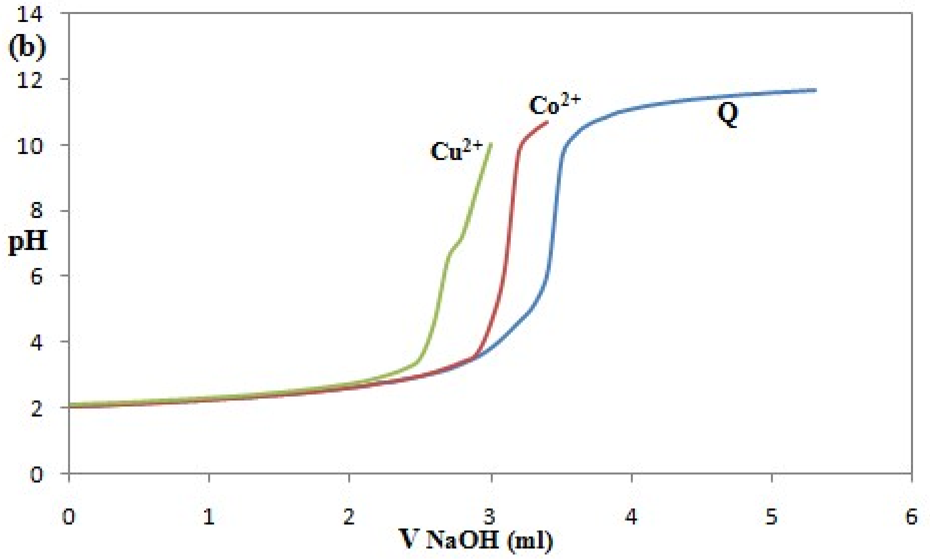

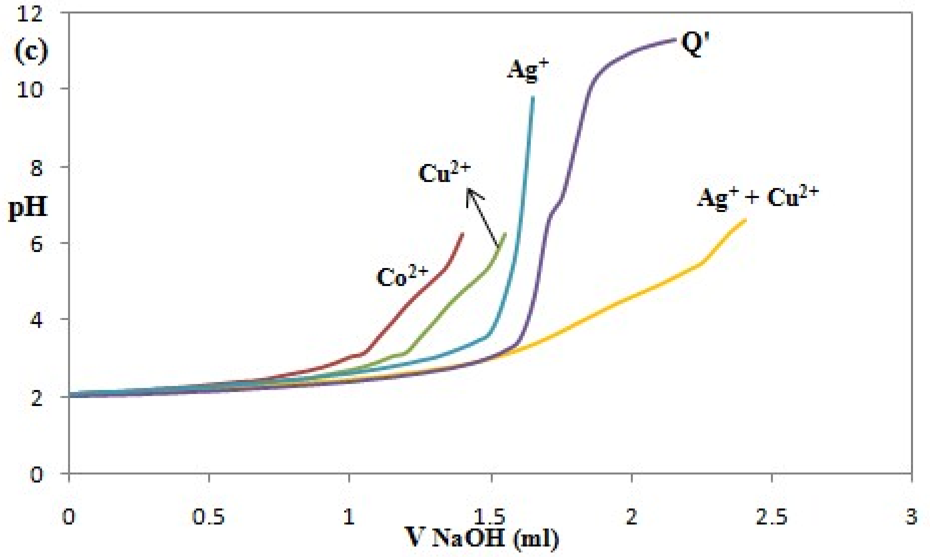

Fig. S21. Potentiometric titration curves of 2,5-pydco (a) and 2,2′-bipyridine (b) and 1,10-phenantroline (c) in the presence and absence of M^{n+} ions with NaOH 0.095 M in aqueous solution at 25 ˚C μ = 0.1 M of KNO_3.
